# Methodological Landscape of DNA Damage Response Detection: From Conventional Assays to Future Innovations

**DOI:** 10.3390/cimb48040339

**Published:** 2026-03-24

**Authors:** Yan Xi, Xinchen Yan, Jiahao Liu, Siqi Li, Xinyang Zhang, Yiwen Hou, Minjie Chu, Minfeng Yang

**Affiliations:** Key Laboratory of Jiangsu Higher Education Institutions for Advanced Medical Analytics and Public Health, Institute for Applied Research in Public Health, School of Public Health, Nantong University, Nantong 226019, China; 15996162317@163.com (Y.X.); thryyxc@163.com (X.Y.); 15862703103@163.com (J.L.); 2317320009@stmail.ntu.edu.cn (S.L.); 2331320092@stmail.ntu.edu.cn (X.Z.); 15851201052@163.com (Y.H.); chuminjie@ntu.edu.cn (M.C.)

**Keywords:** DNA damage response, DNA damage repair, DNA damage detection methodologies, high-throughput screening, biomarkers

## Abstract

All living organisms possess a DNA damage response (DDR) that is important for genetic evolution. Cells have developed comprehensive mechanisms for addressing DNA damage, collectively called the DNA damage response and repair. External environmental stress continuously disrupts genomic integrity and triggers various pathological changes. The failure of the DDR network often drives cell carcinogenesis, and its core components not only serve as biological markers for disease monitoring but also represent highly promising molecular targets for targeted therapy. Therefore, there is a high level of interest in exploring DDR-related biomarkers as cutting-edge therapeutic regimens and developing highly sensitive tools for DDR diagnosis. These methods should assess the rate of damage occurrence and distinguish when repair pathways are activated. These kinds of advances are key to preserving genetic stability and detecting and preventing diseases early. Here, we provide a broad summary of recent advances in DDR detection technologies, with a particular focus on the complementarity between different techniques. We have also summarized current technological bottlenecks, future research paradigms, and clinical translation pathways. The insights presented in this review will contribute to the development of multidisciplinary integrated DDR detection technologies, promote the establishment of DDR biomarker detection systems, and provide crucial methodological references for targeted drug development, efficacy evaluation, and resistance mechanism research targeting the DDR pathway.

## 1. Introduction

The DNA damage response (DDR) is a core mechanism for maintaining genomic stability [1,2]. Defects in this mechanism often drive cancer development, organism aging, and various genetic abnormalities. This highly conserved mechanism constantly monitors and responds to dual threats [3], including external environmental radiation such as ultraviolet (UV) rays, as well as internal reactive oxygen species (ROS) and replication stress-induced damage [4]. In the face of crises, cells will initiate a cascade of signals to forcibly intervene in the cycle process and trigger irreversible apoptosis and senescence programs when conventional repair fails [5]. Clinical medicine is attempting to transform this survival mechanism into an attacking weapon. Targeted drugs such as PARP inhibitors have achieved precise killing by exploiting the inherent repair defects of cancer cells [6]. The real clinical challenge lies in the extremely high heterogeneity of the DDR network in patients’ bodies, which leads to significant differences in drug responses among individuals. This forces researchers to break away from the limitations of a single target and search for new molecular intervention strategies.

## 2. DNA Damage Mechanism

### 2.1. Sources and Types of DNA Damage

**Figure 1:**
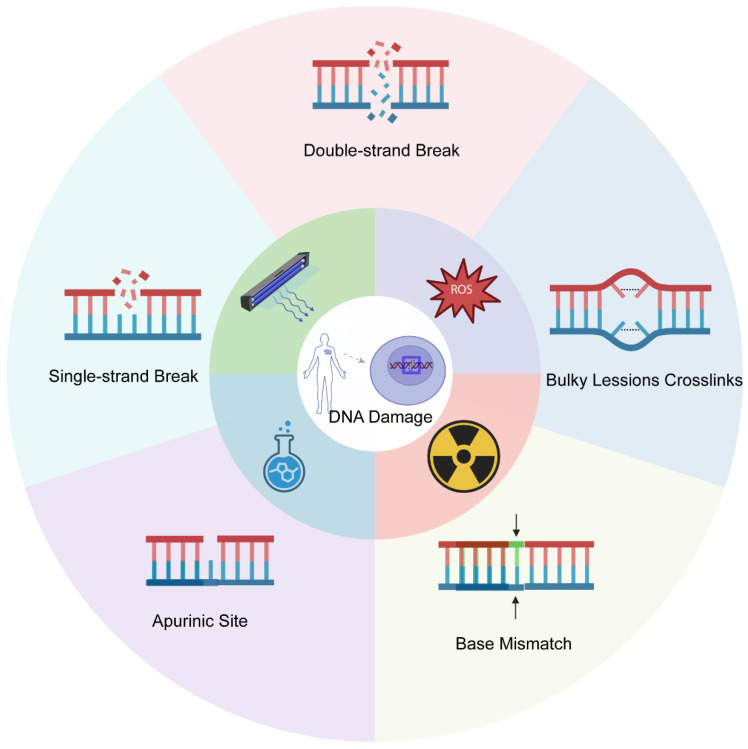
Various endogenous and external stimuli induce distinct types of DNA damage. The primary categories of DNA damage and the endogenous and exogenous factors responsible for inducing it. Some scientific illustrations of the figure were created in BioRender. Xi, Y. (2026) https://BioRender.com/n6ao86s (accessed on 26 February 2026).

DNA is perpetually subjected to damage from both endogenous causes (e.g., ROS, replication stress) and exogenous environmental influences (e.g., ionizing radiation, UV rays, chemotherapeutics). The diverse endogenous and exogenous chemicals that interact with the human body and the potential DNA damage that may ensue are shown in Figure 1. These factors cause various structural lesions by direct chemical bond breakage [7,8] or indirect mediation (e.g., free radical-induced replication fork collapse) [9]. Prevalent lesions encompass double-strand breaks (DSBs), single-strand breaks (SSBs), abasic sites, base mismatches, and macromolecular cross-links. Experimental quantitative data indicate that, whereas SSBs and base losses occur often, DSBs and complex aggregative damages are less common but significantly harmful; inadequate repair of these damages propels the cell towards terminal apoptosis or neoplastic transformation [10].

### 2.2. Biological Consequences of DNA Damage

Dysregulation of the DDR occurs in many diseases, for instance, vascular diseases, ischemic stroke [11], and cancer [12]. When proper DNA damage repair fails, it can negatively impact normal cells. For example, DNA damage-induced autophagy profoundly influences cell fate by maintaining genomic stability and determining survival or death [13]. While such complex DNA damage unequivocally elevates mutagenic potential and cellular lethality, its paradoxical roles in both oncogenic transformation and potential therapeutic applications (the “friend-or-foe” dichotomy) remain unresolved.

## 3. DNA Repair Mechanism

**Figure 2:**
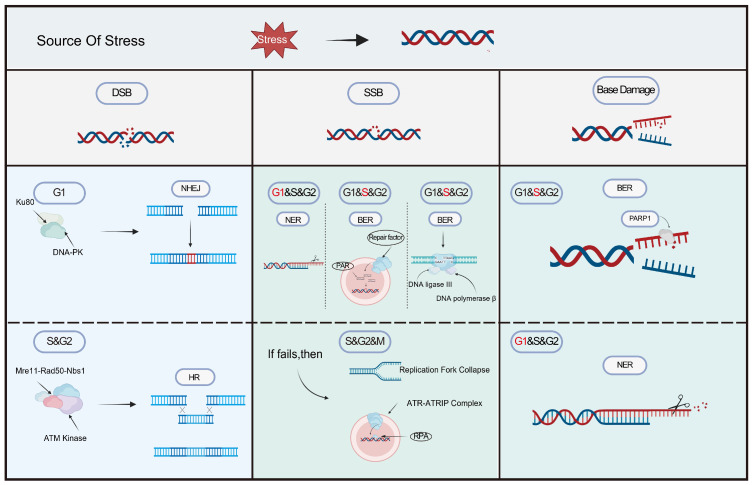
There are various methods available for repairing DNA damage. Different parts of the cell cycle, different types of DNA damage, and different ways that DNA damage can be fixed. Some scientific illustrations of the figure were created in BioRender. Xi, Y. (2026) https://BioRender.com/n6ao86s (accessed on 26 February 2026).

Cells initiate particular repair pathways to maintain genomic integrity, which are precisely regulated according to the kind of DNA damage and the phase of the cell cycle (Figure 2; Table 1). Upon damage detection, checkpoint kinases (such as ATM/ATR and Chk1/Chk2) swiftly initiate cell cycle arrest during the G1/S, S, or G2/M transition phases, providing time for repair [14].

### 3.1. Double-Strand Break Repair

DNA DSBs are predominantly repaired via two mechanisms. Non-homologous end joining (NHEJ) is a fast, template-independent, and less precise method capable of addressing any type of DSB, mostly active during the G1 phase of the cell cycle [15]. Conversely, homologous recombination repair (HRR) utilizes sibling chromatids as templates to ensure high-fidelity repair. HRR is exclusively confined to the S and G2 phases, as sister chromatids are generated following DNA replication during the S phase [16].

### 3.2. Mismatch Repair (MMR)

Mismatch repair (MMR) primarily rectifies base mismatches and insertion/deletion loops that arise during DNA replication. Its role is to verify the accuracy of the just replicated DNA [17]. The MMR system can distinguish newly synthesized strands (prone to errors) from template strands (correct) and remove errors on the new strands. It mainly occurs in the late S phase and G2 phase [16].

### 3.3. Nucleotide Excision Repair (NER)

Nucleotide excision repair (NER) is the principal mechanism in mammals for the removal of bulky DNA adducts, including those resulting from environmental carcinogens (e.g., pyrimidine dimers and chemical adduct substances caused by UV rays) to those induced by chemotherapeutic agents like cisplatin. This kind of damage can significantly impede transcription and replication and must be eliminated promptly. It is active throughout all phases, but is especially crucial during the G1 phase [18].

### 3.4. Base Excision Repair (BER)

Base excision repair (BER) serves as the major pathway for repairing small base damages and abasic sites, which are continuously generated through spontaneous depurination and other processes. Given that various lesions, including those from oxidation, alkylation, and hydrolysis, are a continuous endogenous phenomenon, cells necessitate the prompt and adaptable BER mechanism to remain active throughout the cell cycle to safeguard genomic stability [19]. PARP1-mediated DNA damage repair is perpetually active throughout all phases of the cell cycle (G1, S, G2), with its function during the S phase being especially vital [20].

### 3.5. Direct Repair (DR)

Direct repair (DR) primarily rectifies particular forms of DNA damage induced by alkylating chemicals, namely O^6^-methylguanine [21]. This mechanism can precisely identify the damaged sites and directly remove the modified groups using a single enzyme to reverse the damage, without the need to excise or replace nucleotides, thereby restoring the original structure of DNA [22]. This repair mechanism continues throughout the complete cell cycle.

**Table 1:** Different Characteristics of DNA Repair Pathways.

DNA Repair Pathways	Targeted Lesions	Key Enzyme\Protein	Specificities	Reference
DR	Simple chemical modifications, e.g., base alkylation	MGMT (O^6^-methylguanine-DNA methyltransferase)	Direct reversal of damage without excision of bases or nucleotides	[21]
BER	Minor base damage (oxidation, alkylation, etc.)	DNA glycosylase (e.g., UNG) APE1 DNA polymerase beta	Repairing single base damage and maintaining genomic micro	[23,24]
NER	Bulk damage (e.g., UV induced pyrimidine dimers, chemical adducts)	XPAXPG TFIIH complex ERCC1	Excision of damage containing oligonucleotide fragments (~2432 nt)	[25,26,27]
MMR	Base mismatches due to replication errors (e.g., GT), IDLs	MutSα MutLα	Repairing replication errors and reducing spontaneous mutation rates (defects leading to Lynch syndrome)	[28,29]
HR	DSB Replication fork collapse	MRN complex MRE11RAD50NBS1 RAD51	High fidelity restoration, acting only on S/G_2_ phase	[30]
NHEJ	DSB	Ku70, Ku80, DNA-PKcs	Rapid but indel-prone	[31,32,33]

Abbreviation: DR, direct repair; MGMT, methylguanine methyltransferase; BER, base excision repair; APE1, AP endonuclease; NER, nucleotide excision repair; MMR, mismatch repair; MutSα, MSH2MSH6; MutLα, MLH1PMS2; IDLs, insertion/deletion loops; HR, homologous recombination; DSB, DNA double-strand break; NHEJ, nonhomologous end joining.

## 4. Strategies for Assessing DDR

The DDR is not an isolated single reaction but rather a highly coordinated biological network. This system precisely regulates cell division and integrates damaged genes and will promptly issue a death command when damage overload occurs [34]. This mechanism is the final line of defense against harmful mutations, and its malfunction often directly drives cancer or brain diseases [1]. Unveiling the underlying laws of this network actually amounts to obtaining a new key for disease intervention [35,36]. Facing such a high biological cost, the core demand of the laboratory has shifted to precisely capturing these fleeting repair dynamics. The scientific community has thus constructed a multi-dimensional detection system, and each underlying technology provides a unique observation window for analyzing the DDR network.

### 4.1. Traditional Molecular Biological Methods

The traditional molecular detection toolkit, with its low cost and high reproducibility, remains the preferred choice for basic experiments to this day. Researchers must carefully select the approach based on the target when designing experiments: is it to capture the macroscopic characteristics of the population, focus on the detailed analysis of single cells, or simply conduct qualitative screening [37,38]? The classic methods are actually very reliable in low-throughput validation, but when facing the complex DDR global network, their lack of a macroscopic perspective becomes completely exposed. Only by deeply integrating these classic methods with modern multi-omics technologies can the blind spots of a single perspective be completely eliminated.

#### 4.1.1. Analysis of Damage Based on Microscopic Observation and Protein Localization

Long before the advent of high-throughput sequencing technology, direct observation of cell morphology enabled us to recognize that changes in cell shape and protein distribution were the basis for studying DNA damage. These optical techniques give researchers visual proof, translating abstract molecular events into tangible visual evidence. However, this clarity comes at a cost: in practical laboratory settings, we often wrestle with the trade-off between the depth of visual detail and the breadth of sample throughput. Furthermore, while a microscope captures a snapshot of cellular distress, it often struggles to convey the full, dynamic movie of the repair process. Although certain morphological observations have a degree of subjectivity, classic methods such as chromosome aberration analysis and micronucleus tests have been standardized and quantitatively analyzed through the establishment of strict international guidelines (such as the organization for economic cooperation and development [OECD] guidelines), thereby effectively overcoming this limitation.

##### Analysis of Chromosomal Aberrations

Chromosome aberration analysis is a classic method that directly observes the chromosomes of cells when they are at the metaphase stage under a microscope. This method reveals physical breaks or missing pieces [39]. It provides direct visual evidence of genomic instability caused by DDR defects and is one of the gold standards in the fields of genotoxicity, tumor genetics, and clinical diagnosis. Its advantage lies in its unparalleled intuitiveness, which enables the identification of specific aberration types, which is crucial for mechanism research. Moreover, its scientific validity and regulatory importance have been long confirmed, and independent international testing guidelines have been formed. For example, the testing guidelines of the OECD TG 473 (in vitro mammalian cell chromosome aberration test) and TG 475 (in vivo chromosome aberration test using mammalian bone marrow cells) specifically regulate this method, emphasizing its unique role in evaluating genotoxic agents [40]. These guidelines provide descriptions of the experimental system, ensuring the results from different laboratories are comparable. However, this detection method relies on cells entering the mitotic stage and usually requires in vitro culture or collection of samples from the body, followed by mitotic blockage treatment. The efficiency of this treatment is low, the process takes a long time, and it relies on specialized cell culture and chromosome analysis techniques. The analysis of chromosomal aberrations reflects relatively severe chromosomal damage fixed after a complete cell cycle has been completed in the cells and might miss early repairable DNA damage signals.

##### Micronucleus Test

The micronucleus test is a classic cytogenetic method that assesses chromosomal integrity damage and genomic instability by detecting the formation of micronuclei in interphase cells [41]. Micronuclei are small nuclear-like structures outside the main nucleus, containing entire lagging chromosomes or chromosome fragments without centromeres, which result from errors in chromosome segregation during the late stage of cell division or after DNA breaks [42]. Micronuclei are formed by exposure to genotoxic substances in certain human cells, including lymphocytes and buccal mucosa cells [43]. The scientific validity and regulatory importance of the micronucleus test have been widely recognized; for example, the testing guidelines of the OECD TG 487 (in vitro mammalian cell micronucleus test) and TG 474 (in vivo mammalian erythrocyte micronucleus test) specifically regulate this method. This makes it clearly distinguishable from chromosome aberration analysis (OECD TG 473/475) in the regulatory framework, together forming a complementary genetic toxicity testing combination [40].

The high standardization of its methodology makes it one of the core tools in genetic toxicology, biological monitoring, and cancer risk assessment [44]. In vitro tests typically use the cytoplasmic division blocking method to specifically analyze cells that have completed one mitotic division, significantly improving the accuracy and reproducibility of the results. Compared to chromosome aberration analysis, its advantages lie in being relatively simple in technology, rapid in scoring, and straightforward to combine with flow cytometry (FACS) or high-content imaging systems for high-throughput analysis [45,46]. It is applicable to various cell types (such as lymphocytes), and the acquisition of some samples has a non-invasive characteristic, making it convenient for biological monitoring in population epidemiological studies [47].

##### Immunofluorescence (IF)

Immunofluorescence (IF) was one of the earliest and most widely used techniques for the identification and localization of some proteins in cells, especially to obtain comprehensive conclusions in complex mechanisms [48]. Researchers used IF to observe the dynamic behavior of key repair proteins in real time at the single-cell, single-allele level. Researchers can also use IF to find and measure damage and responses in tissue sections, which lets them look at how the same DNA damage can cause different biological effects in different organs [49,50]. This technique provides unique advantages for investigating DDR dynamics at the single-cell resolution. IF helps scientists see how DDR parts are organized in space and time. While IF achieves high sensitivity in detecting focal DDR signals, its specificity is dependent on antibody validation, and nonspecific background can compromise accuracy in dense nuclear regions. Technical variables such as positioning efficiency and epitope accessibility may affect the quantitative performance. Western blot analysis is a necessary way to check the levels of protein expression quantitatively. High-throughput sequencing methods, like RNA-seq, can reveal how molecular mechanisms work [51]. Combining these methodologies enables researchers to draw more conclusions about DDR in a biological way.

#### 4.1.2. Quantitative Analysis of Proteins/Nucleic Acids Based on Molecular Separation and Hybridization

These detection techniques utilize electrophoresis separation, specific hybridization, etc., to conduct semi-quantitative or quantitative analysis of protein or nucleic acid targets. Their advantages lie in high specificity, mature technology, and the ability to detect post-translational modifications (such as phosphorylation), but they cannot retain spatial information and are difficult to meet the requirements of high-throughput screening (HTS).

##### Immunoblotting

Immunoblotting is an indispensable tool in DDR research, particularly suitable for comprehensive analysis of complex signaling pathways. This technique utilizes specific antibodies to detect specific DNA damage markers (such as γ-H2AX, p-ATM, and p53). Its advantage lies in the high specificity and low cross-reactivity of the antibodies [52].

It is not only applicable for detecting protein-level cascades of modifications caused by various DNA damages, including responses related to DSBs and oxidative damage, but also capable of semi-quantitative analysis of the total signals of these phosphorylation events at the population level [53]. With this technique, researchers have discovered that persistent DNA DSBs can activate the ATM/NBS1/CHK2 signaling pathway (rather than p53), thereby triggering the secretion of senescence-related inflammatory factors [54]. This indicates that the DDR coordinates cell-autonomous reactions such as repair and has a non-autonomous communication function of transmitting damaged signals to the surrounding microenvironment, providing a new perspective for understanding the correlations between ageing, cancer, and inflammatory diseases [55]. Although this technique has high cost-effectiveness, its results depend highly on the quality of antibodies. Non-specific binding is prone to cause false positives and is difficult to achieve absolute quantification [56]. Moreover, immunoblotting has a lower throughput compared to high-throughput omics technologies, and it can only detect a small number of samples in each experiment. At the same time, as the detection relies on cell lysates, this method cannot provide spatial localization information of the damage within the cell nucleus. To overcome these limitations, combining with methods like immunoprecipitation can enhance the accuracy and depth of analysis to a certain degree.

##### Analysis Based on PCR and Sequencing

Analysis based on PCR and sequencing is suitable for gene mutation detection and DDR target analysis [57]. Its advantage lies in the discovery of unknown mutations, structural variants, or novel regulatory mechanisms (e.g., whole genome sequencing [WGS]). It can accurately identify the types of single-base mutations (such as C > T transitions and deletion sites) [58,59]. The analysis methods based on PCR and sequencing can perform multi-dimensional analysis. For example, WGS can detect whole-genome damage, such as chromosome breakage, while RNA-seq can reveal changes in transcriptional regulation, including the activation of the p53 pathway. And when combined with Unique Molecular Identifier (UMI) technology, it avoids the amplification bias of PCR and has high specificity [60]. Its downsides are also obvious. Data analysis requires bioinformatics support (e.g., mutation annotation and pathway enrichment), which is expensive and complex and time-consuming to store and process. Low-frequency mutations require high-depth sequencing, and some lesions (e.g., DNA cross-linking) may be difficult to capture [61].

As extensions of PCR, quantitative PCR (qPCR)/reverse transcription PCR (RT-PCR) are powerful tools for analyzing expression changes in DDR genes (e.g., *BRCA1*) [62]. qPCR/RT-PCR provides elevated sensitivity and specificity for identifying low-abundance transcripts, quantitative reproducibility, expedited HTS, and suitability for constrained samples in patient biopsies [63]. These methods also allow correlation of gene expression with functional outcomes, e.g., reduced homologous recombination efficiency upon *RAD51* suppression [64]. qPCR/RT-PCR possess an inability to directly assess protein activity or post-translational modifications [65]. At the same time, there are challenges in primer design due to pseudogenes or splice variants [66] and a lack of mechanistic insight [67]. Results can be affected by RNA quality, reverse transcription efficiency, and normalization methods [68]. The technique is restricted to known targets, unlike RNA-seq, which can identify novel DDR genes. qPCR/RT-PCR plays a significant role in the analysis of DDR gene expression. By combining protein detection and genomic techniques (such as RNA-seq), a comprehensive understanding of the mechanism can be obtained [69].

#### 4.1.3. Direct Detection of DNA Damage Based on Electrophoresis

Electrophoresis tests the physical integrity of DNA. It applies an electric field to pull DNA molecules through a matrix. It can be conducted at the single-cell level in an intuitive manner, and its cost is not high. However, distinguishing the types of damage is difficult, the results are susceptible to operational effects, the throughput is low, and severe DNA breaks (such as those occurring in the late stage of apoptosis) can interfere with result interpretation.

##### The Comet Assay

The comet assay (single-cell gel electrophoresis) is a widely used method for assessing nuclear DNA damage in individual eukaryotic cells from yeast to humans. It detects strand breaks and alkali-labile sites (e.g., apurinic/apyrimidinic sites) [70]. Its sensitivity is highly dependent on experimental conditions [71], leading to variability across laboratories. Additionally, its specificity is uncertain. For example, without the use of additional enzymes or program modifications, the assay cannot reliably differentiate SSBs, base sites, and repair intermediates [72].

However, the assay’s reliability is well-established through extensive validation by academic, industrial, and regulatory researchers. The OECD has approved it as a standardized in vivo test for checking DNA damage in animal tissues because it is useful for testing genotoxicity [73]. This endorsement came after a lot of collaborative work, including the first multi-laboratory review on the comet assay, published in 1993 [74], the first effort to make comet assay guidelines for genetic toxicology, published in 2000 [75], and formal validation studies conducted between 2006 and 2012. The OECD officially accepted the in vivo mammalian alkaline comet assay as part of test guideline 489 in 2014, and it was updated in 2016 [76].

Compared to alternative genotoxicity tests, the comet assay provides superior resolution at the single-cell level, though it lacks the throughput and standardization of micronucleus or γH2AX assays [77]. Few systematic reports provide quantitative performance indicators, and false positives can occur due to apoptosis or necrosis [78].

While the assay is less costly and time-consuming than chromosomal aberration tests, variability in scoring and imaging systems affects reproducibility [79]. For HTS, an automatic comet analyzer can be used. By combining the comet detection method with fluorescent probes or enzymatic treatments, the types of damage can be clarified for mechanism research.

### 4.2. Contemporary Detection Methods of DDR

Contemporary DDR detection technologies offer new perspectives by shifting toward automation and single-molecule tracking. These cutting-edge techniques provide exceptional sensitivity at single-base resolution. However, their high cost and complicated operation restrict their broad application.

#### 4.2.1. High-Throughput and Sequencing Technologies

These technologies aim to make molecular maps of the whole genome. Their advantages include analyzing molecular networks. However, their high costs and the complexity of analyzing the data are still problems that need to be solved.

##### Chromatin Immunoprecipitation Sequencing (ChIP-seq)

Chromatin immunoprecipitation sequencing (ChIP-seq) is a powerful tool that can be used to map the binding sites of transcription factors related to DNA repair such as p53, γ-H2AX, and BRCA1, as well as the histone modifications throughout the entire genome. It offers genome-wide insights into DDR regulatory mechanisms [80]. Benefits of ChIP-seq include identifying precise binding loci, detecting novel regulatory elements involved in DDR, and revealing dynamic changes in protein-DNA interactions after DNA damage [81]. ChIP-seq has some excellent features, but it also has some drawbacks, for example, high input material requirements, which may be challenging for clinical samples. It may also have the problem of false positive results due to its reliance on antibody specificity, and it cannot directly display the identified binding events [82]. For instance, post-injury sampling is frequently employed to study epigenetic or transcriptomic alterations directly induced by treatments such as drugs, viral infections, or environmental stress. The timing of sampling is critical. Premature sampling fails to capture sufficient changes, while late sampling can only reveal secondary effects. The sampling time must be determined based on pre-experiments or existing literature [83]. Additionally, choosing the appropriate control group is also crucial, such as required input DNA control and IgG control groups, etc.

ChIP-seq requires a complex bioinformatics process for data analysis and appropriate controls to distinguish the results. ChIP-seq is more costly and computationally demanding than methods such as qPCR, but it has a wider coverage than protein-centered techniques (such as immunoblotting). The combination of ChIP-seq with other omics methods (such as RNA-seq and ATAC-seq) has been proven valuable for a comprehensive understanding of DDR regulatory networks [84]. If combined with RNA-seq, it can closely link the binding events with the transcriptional results; if combined with ATAC-seq, it can explore the relationship of chromatin accessibility. This integration approach is the most effective [85]. For example, combining ChIP-seq with RNA-seq helps to clarify whether transcription factors or histone modifications directly regulate gene expression [86].

##### RNA-seq and Single-Cell RNA Sequencing (scRNA-seq)

Single-cell RNA sequencing (scRNA-seq) can comprehensively analyze the gene expression profiles of cell populations at the single-cell resolution [58,87]. This technology helps identify new genes and pathways related to DDR and precisely interpret the heterogeneity of DDR activation in complex tissues or tumors [88]. By analyzing the expression levels of DDR-related genes in different cell types [62], scRNA-seq can efficiently identify cell subpopulations with specific DNA repair capabilities [89]. However, this technology has obvious limitations: the observed transcriptional level changes may only reflect the secondary stress responses of cells rather than direct DDR activation; at the same time, key post-translational modification events (such as ATM phosphorylation or γ-H2AX focus formation) cannot be directly captured [90]. Furthermore, due to the destructive nature of the detection process on the samples, scRNA-seq is difficult to longitudinally track the dynamic changes in DDR in individual cells [91]. Compared with conventional RNA-seq, scRNA-seq sacrifices a certain sequencing depth and has a higher cost per single cell. However, it provides higher cell resolution at the cost of sacrificing sequencing depth. To compensate for this defect, when scRNA-seq is combined with methods such as phosphorylation FACS (proteomics), the transcriptomic data can be correlated with protein-level events for analysis.

#### 4.2.2. Large-Scale Functional Genomics Screening Technology

This type of technology identifies key factors or drugs that affect DDR by testing a large number of compounds or altering genes. It stands out among various detection methods due to its high throughput and rapid ability to discover new synthetic lethal pairs. The main limitations of these technologies lie in their high cost, high false positive rate, and the differences between the screening conditions and the physiological environment.

##### High-Throughput Screening (HTS) Technology

High-throughput screening (HTS) technology has accelerated the progress of DDR research by enabling comprehensive genomic analysis and expedited drug screening, thereby enhancing the efficiency of identifying critical DDR components and potential therapeutic targets [92]. The advantage of HTS in DDR research lies in its ability to simultaneously and systematically test thousands of genes or chemical substances, accelerating the discovery of new DDR-related genes and DNA repair regulatory factors [93]. HTS is especially useful for finding synthetic lethal interactions in cancers that don’t have DDR (like how *BRCA* mutant cells are sensitive to *PARP* inhibitors) [94]. However, there are some limitations to HTS methods, such as the need for extensive validation to reduce the high false positive rate. Additionally, under artificial screening conditions, to reduce costs, the complex DDR pathways may be simplified [95]. Although HTS can identify DDR-related candidates, understanding the biological relevance of these findings requires subsequent mechanistic studies [91]. To make up for the subsequent biological relevance demands, the integration of HTS with other technologies has enhanced its utility in DDR research by providing more comprehensive insights into DNA repair mechanisms and potential therapeutic vulnerabilities, for instance, using CRISPR libraries containing tens of thousands of gRNAs to target genes throughout the entire genome. Then, the library was introduced into cell lines sensitive to DNA damage (such as cells lacking *p53*), and the cells were treated with DNA damage agents (*PARP* inhibitors, olaparib, etc.). The genes that have been knocked out in the surviving cells are very likely to be crucial for resisting this DNA damage. Then, by analyzing the distribution of gRNA in surviving cells through high-throughput sequencing and comparing it with the initial library, those genes that “give cells a survival advantage after knockout” can be identified [96].

##### CRISPR-Cas9 In Vitro Gene Screening

The CRISPR-Cas9 gene screening technology enables the systematic discovery of DDR-related genes and potential therapeutic targets by knocking out the target genes in vitro [97,98]. As a high-throughput technique, it allows researchers to conduct genome-wide functional screening and precisely identify known and new components of the DDR pathways [99]. At the same time, the accuracy of CRISPR editing is helpful in establishing clear correlations between genotype and phenotype, which is particularly important for identifying synthetic lethal interactions in DDR-deficient cancer cells (such as the sensitivity of *BRCA*-deficient tumors to *PARP* inhibitors) [100,101]. Although the specificity of modern CRISPR systems has significantly improved, the off-target effects still need to be verified through protein-level analysis (such as immunoblotting) [102]. Additionally, complete knockout often leads to the complete loss of the target gene’s function, which may result in the omission of subtle phenotypic changes that occur when some genes are inactivated; the in vitro screening environment may also not fully simulate the regulatory mode of the DDR pathway in vivo [103]. Compared with RNAi screening, the gene knockout efficiency of CRISPR is more stable, but it is more costly and the library design is more complex [104]. Compared with chemical screening, it can identify gene dependencies, but it cannot directly target the regulation of protein interactions [105]. Despite the significant screening efficacy of this technology, it usually requires subsequent studies to elucidate its molecular mechanism [106]. To overcome these limitations, when combined with transcriptomics or proteomics techniques, CRISPR can effectively link gene discovery to its molecular function [107].

#### 4.2.3. Dynamic and Real-Time Monitoring Technology

This type of technology enables real-time, dynamic, and quantitative detection of the DDR process in living cells. Its core advantages lie in providing unparalleled temporal resolution and kinetic information. The limitations of these technologies lie in their high requirements and the possibility of causing phototoxicity that could interfere with normal physiological processes.

##### Real-Time Reporter Gene Systems

Real-time reporter gene systems can detect the spatial and temporal dynamic changes in DDR in living cells. By integrating fluorescent proteins (e.g., GFP, mCherry) or luciferase-based reporter gene sequences with the gene sequences of DDR components (such as *p53*, *ATM*/*ATR* kinases, and *NBS1*) [108], researchers can track the localization, recruitment, and activation of repair factors in real time [109]. Although this system can visualize the dynamic assembly of repair focal points, the propagation of damage signals, and the coordination of different DDR pathways, it has high requirements for experimental techniques. Researchers fuse the coding sequence of the fluorescent protein with the N-terminal or C-terminal of the target protein to express the fusion protein. Even though many proteins can accept this fluorescent labeling, the procedure may still affect the folding or function of the protein, so control experiments are needed to confirm that the behavior of the fusion protein is consistent with the endogenous protein. In addition, overexpression of the labeling component may interfere with the cellular process being studied. Therefore, to ensure that overexpression does not artificially enhance DDR signal transduction, it is necessary to prove through functional restoration experiments that the labeled protein can replicate the endogenous function. Compared with fixed-cell imaging, real-time reporter gene systems can provide kinetic data, but they require higher equipment costs and computing resources. Compared with biochemical assays, real-time reporter gene systems retain the cellular environment but lack the molecular precision for detecting post-translational modifications other than the labeling components. To obtain a deeper understanding of the mechanism, real-time reporter gene system data should be combined with complementary methods (such as phosphorylation proteomics or scRNA seq) to determine the physiological relevance of the observed localization patterns.

##### Super-Resolution Microscopy

Super-resolution microscopy has surpassed the diffraction limit of traditional optical microscopes. This method enables the resolution and reconstruction of structural details of the focal point. It also tracks the dynamic process of DDR-related factors recruited under genotoxic stress conditions [110]. This technique utilizes fluorescently labeled immunoconjugates with specific photophysical properties [111] for detecting antibody-protein complexes. Using super-resolution microscopy, DDR proteins like γ-H2AX (a marker of DNA DSBs) and RAD51 (a recombinase in homologous recombination) can be observed to move around and attach to DNA damage sites [112]. Its single-molecule localization methods include Photoactivated Localization Microscopy (PALM) and Stochastic Optical Reconstruction Microscopy (STORM). The two achieve imaging beyond the diffraction limit through the random activation of sparse fluorescent molecules. This method can directly visualize individual repair protein complexes and provides quantitative nanoscale insights into the dynamic process of DSB repair. Compared to traditional microscopes, super-resolution microscopy has significantly improved spatial resolution, but its equipment cost and required special reagents are much higher. Compared to biochemical methods, it can retain spatial background information but lacks high-throughput statistical analysis capabilities. Super-resolution microscopy excels in describing the nanoscale organization of repair foci and the molar ratio of protein complexes but is not suitable for high-throughput compound screening or long-term dynamic studies of live cells sensitive to light toxicity. This technique often requires fixation of samples, and using a gentler fixation method (such as using paraformaldehyde under optimized conditions) it can reduce the artefacts caused by fixation. Combining microscope observations with functional assays can further determine the biological relevance of the observed nanoscale localization patterns [113,114,115].

#### 4.2.4. High-Throughput Phenotyping and Single-Cell Analysis Techniques

These technologies can rapidly and comprehensively analyze the heterogeneity of cell populations or single cells. Their advantages lie in their high speed and numerous parameters. However, the problem of reducing costs in high-throughput conditions is currently difficult to overcome, and some technologies may lose spatial information.

##### Flow Cytometry (FACS)

Flow Cytometry (FACS) detects the population of γ-H2AX-positive cells for analysis of DDR [116,117]. This technique can rapidly and high-throughput quantitatively measure the DNA damage levels of millions of cells. Meanwhile, it simultaneously measures multiple parameters through multi-color fluorescence labeling, including cell cycle status, apoptotic markers, and other proteins related to DDR. These enable researchers to correlate the severity and heterogeneity of DNA damage with specific cell cycle stages or other functional outcomes in the population [118]. Compared to microscopy-based methods, FACS can handle a large number of cell samples and can be used for fixed and live cell analysis when using fluorescent DDR reporters [119]. However, this method still has limitations. It lacks the spatial resolution of individual cell nuclei for DNA damage foci provided by microscopy, thus potentially missing important subnuclear localization patterns of DDR factors. Additionally, it cannot analyze DDR in intact tissue environments due to the need to prepare single-cell suspensions. And this technique cannot provide information on the specific type of DNA damage or genomic location. Compared to imaging techniques, FACS offers stronger statistical analysis capabilities and higher throughput, but it cannot capture the spatial distribution patterns of DDR factors. Compared to molecular methods such as immunoblotting, it maintains single-cell resolution but lacks protein size validation capabilities and requires careful fluorescence compensation to avoid spectral overlap. Although FACS data has high value for large-scale DNA damage repair assessment, it usually requires supplementary techniques such as IF microscopy or protein blotting for validation to confirm specific DNA repair activation patterns and mechanisms [120].

##### Cell Viability Assay

Cell viability assays provide quantitative methods for assessing the impact of DDR defects on cell survival [121]. These colorimetric or luminescent assays can screen cell viability rapidly and with a high throughput under various experimental conditions. These enable efficient comparison of the sensitivity of different cell lines to different DNA damage agents (for example, normal DDR function vs. defective DDR) [122]. They are suitable for identifying synthetic lethal interactions in DDR-defective cells and for preclinical evaluation of DDR-targeted anticancer drugs [123]. These cell viability assays still have some limitations. They cannot directly detect the specific DDR pathways affected or reveal the molecular mechanisms underlying the observed survival changes [124]. Moreover, these detection methods cannot distinguish between different cell death modes caused by DNA damage (apoptosis, necrosis, senescence). Also, they cannot detect subtle phenotypic changes that occur before the loss of viability [125]. Additionally, the readings of metabolic indicators may be affected by factors unrelated to DNA repair capacity, such as changes in proliferation rate or mitochondrial function [126]. Although these assays have high sensitivity in detecting overall changes in cell viability and provide cost-effective, HTS capabilities, their phenotypic specificity is low. For example, simple metabolic indicator readings (such as MTT and ATP) cannot distinguish DNA damage-specific effects from general cytotoxic stress responses, nor can they distinguish between apoptosis and necrosis or senescence. Moreover, their resolution is limited to endpoint indicators at the population level and lacks temporal dynamics or molecular mechanism insights at the single-cell level. Therefore, the best use of these assays is as a primary screening tool to identify candidate conditions that require further molecular analysis, rather than as independent evidence to elucidate the DDR mechanism [127]. Compared to clonogenic survival assays, viability tests are faster but less applicable for evaluating long-term proliferation ability. Cell viability assays typically require subsequent experiments using more specific DDR markers and mechanism studies to fully interpret their biological significance.

##### Cell Function Tests

Cell function tests can directly assess the response of cells to genotoxic stress [128,129]. Specifically, imaging-based methods can track the temporal changes of key events in the DDR (such as γ-H2AX, p53, or RAD51 focal formation) through fluorescent reporter genes or IF labelling, thereby differentiating between acute and persistent DDR [98]. Functional tests based on FACS can quantitatively analyse DDR markers in thousands of single cells to reveal the population heterogeneity of the damage response [130]. However, these methods all have significant limitations: for instance, many functional tests based on exogenous expression require genetic modification (such as constructing reporter cell lines), which may interfere with the natural regulation of the DDR pathway and the difficulty of fully simulating the complex microenvironment of damaged tissues in vivo in vitro culture conditions [131]. Moreover, for non-imaging methods such as FACS, their resolution is usually limited to the population level or the overall fluorescence intensity of a single cell, unable to resolve the subcellular localisation of DDR factors, and most lack molecular specificity for different types of DNA damage. Compared to endpoint molecular detection, live cell methods can provide more abundant dynamic information, but they usually require higher equipment costs and more complex reporting systems [132], and their sensitivity largely depends on probe design and detection threshold setting. Although cell function tests have advantages in phenotypic observation, due to their difficulty in directly revealing the underlying mechanisms, they usually need to be combined with molecular techniques such as immunoblotting or sequencing to establish a complete mechanism correlation [133].

##### Direct Detection of the Activity of Key Repair Enzymes

Direct detection of the activity of key repair enzymes focuses on detecting the catalytic states of DDR signaling kinases and other key enzymes, providing direct evidence for the activation of key repair enzymes [134]. The most commonly used alternative method for monitoring kinase activity is to detect its post-translational modifications, especially the phosphorylation level [135]. For example, ATM (ataxia-telangiectasia-mutated protein) and ATR (ATM and Rad3-related) are rapidly activated in response to different types of DNA damage [136]. Their activation is manifested as self-phosphorylation and subsequent phosphorylation of numerous downstream substrates. Techniques such as immunoblotting or IF, using phosphorylation-specific antibodies, can detect these key events, such as the phosphorylation of Chk1 at the Ser345 site [137]. Compared with high-throughput gene screening methods, biochemical verification of key repair enzymes can more accurately reflect functional output, but it has a lower throughput and higher operational complexity. Especially when compared with imaging-based methods such as IF, biochemical assays such as immunoblotting provide precise population biochemical quantification but lack spatial and single-cell information. Considerations of these experiments include choosing validated phosphorylation-specific antibodies, setting appropriate positive and negative controls, and standardising with the total protein level. These considerations make sure to distinguish true enzyme activation from changes in protein expression levels. To obtain a comprehensive understanding, enzyme activity data should be combined with complementary methods for evaluating downstream functional consequences, for example, comet assays for detecting DNA repair efficiency or colony formation assays for evaluating long-term survival.

In addition to direct detection of the activity of key repair enzymes, current advanced methods can also achieve direct localization of DNA damage, dynamic proteomics analysis, and precise spatiotemporal control. In terms of damage localization, DNA sequencing technology has reached the level of whole-genome and nucleotide resolution. For example, XR-seq achieves precise damage localization by capturing the oligonucleotide fragments that are excised during the NER process [138]. Guide-seq, on the other hand, maps the distribution pattern of DSBs by tracking the integration sites of double-stranded oligonucleotide probes in the genome DSBs [139]. In the field of dynamic proteomics analysis, proximity tagging technologies represented by TurboID have overcome the limitations of traditional affinity purification methods [140]. By fusing biotinylated enzymes with DDR-related proteins, these technologies can label interacting proteins or adjacent proteins with high spatiotemporal resolution. When combined with quantitative mass spectrometry analysis, researchers can analyze the recruitment and dissociation kinetics of repair factors at the damage site and reveal the assembly sequence of the repair complex. In terms of precise spatiotemporal control, the use of photosensitive proteins (such as KillerRed) can induce local damage through targeted light irradiation to produce ROS. At the same time, chemically or optically induced Cas9 systems allow the introduction of DSBs at specific genomic loci on demand [141,142]. This precise control makes it possible to study repair mechanisms in specific chromatin environments. Although these methods have multi-dimensional analytical capabilities, they also face significant challenges in bioinformatics analysis, as well as high costs and technical barriers.

Different strategies for assessing DDR each have different advantages and disadvantages, which are summarized in Table 2, Table 3 and Table 4.

### 4.3. Methodological Details in the Analysis of Key DDR Markers

While the technologies described above provide the hardware for DDR detection, the accuracy of the biological conclusions relies entirely on the nuanced handling of specific molecular markers. Previous literature often merely lists the markers. Here, we critically evaluate the analysis details of the main DDR markers.

#### 4.3.1. The Gold Standard for DSBs: Quantifying γ-H2AX

The phosphorylation of serine 139 of histone variant H2AX (γ-H2AX) is the gold standard for detecting DSBs, but its analysis requires strict methodological considerations [139]. In IF, counting discrete foci (focus) can provide high sensitivity for low-dose damage, while FACS is used to measure the global nuclear fluorescence intensity [140]. A major experimental defect is the failure to exclude cell cycle-related background signals. During the S phase, undisturbed cells naturally show an increase in γ-H2AX levels due to physiological replication stress. Therefore, to prevent false positives, IF or FACS analysis of γ-H2AX must be combined with cell cycle markers (such as DAPI/PI content) or DNA synthesis analogues (such as EdU incorporation) for multiple detection to exclude S-phase interference [141]. Additionally, apoptotic cells, due to DNA fragmentation, form γ-H2AX rings in the entire nuclear region. It is necessary to distinguish this from true repair foci using morphological characteristics to ensure the accuracy of damage assessment.

#### 4.3.2. Evaluating Repair Pathway Choice: 53BP1 and RAD51

The antagonistic effect between 53BP1 and BRCA1/RAD51 determines the repair choice between NHEJ and HRR. The analysis of RAD51 is highly dependent on IF technology for the visualization of the single-strand DNA (ssDNA) loading process. Methodologically, the analysis of RAD51 (and RPA) in a chromatin-bound state requires a crucial “pre-extraction” step: cells must be treated with a mild detergent (such as 0.1% Triton X-100) before fixation to wash away a large pool of soluble nuclear proteins, thereby revealing the specific chromatin-bound part at the repair site [142]. Moreover, since HRR is limited to the S/G2 phase, isolated RAD51 focus analysis lacks biological significance unless co-stained with Cyclin A or Geminin to confirm the cell cycle stage. In contrast, 53BP1 forms a unique nucleoid in the G1 phase. By analyzing the co-localization or exclusion of the two through super-resolution microscopy, one can obtain direct functional readings of the cell’s repair decision-making process [158].

#### 4.3.3. Capturing Transient Activation: Upstream Kinases and RPA32

ATM and ATR kinases are rapidly activated after damage perception, making them preferred targets for protein immunoblotting and FACS. The methodological challenge lies in the transient nature of their phosphorylation. Sample preparation must use rapid freezing or a rapid lysis buffer containing potent broad-spectrum phosphatase inhibitors to retain the fragile phosphorylated epitopes [159]. Additionally, the hyperphosphorylation of RPA32 (such as at Ser4/8 or Ser33 sites) is a clear sign of extensive end resection and replication catastrophe. Combining EdU pulse labelling with flow cytometric detection of RPA32 phosphorylation can precisely and accurately locate replication fork collapse. Compared to simple viability detection, this method provides deeper mechanistic insights [160].

The main limitation of current DDR research lies in the disconnection between general damage detection and the precise identification of specific repair pathways. To transition from descriptive genetic toxicity screening to mechanism analysis, researchers must use targeted indicators to directly detect different DDR networks. For instance, although the standard alkaline comet assay can detect a wide range of damage types (SSB, DSB, and base instability sites), it cannot identify specific repair pathways [161]. By introducing specific damage recognition enzymes, such as formylaminopyrimidine DNA glycosylase (FPG) or endonuclease III (Endo III), the improved comet assay can become a specific tool for evaluating BER efficiency under oxidative damage. Similarly, non-programmed DNA synthesis (UDS) detection is a qualitative functional test for NER, which achieves this by measuring the amount of nucleotide analogue incorporation during the large adduct repair process induced by UV radiation outside the S phase [162].

#### 4.3.4. Assessing Functional Competence: DR-GFP and EJ5-GFP Reporters

Although it is crucial to detect general damage markers such as γ-H2AX, these markers cannot determine whether cells use the HRR or NHEJ repair pathways. To address this issue, pathway-specific functional reporters, particularly the DR-GFP and EJ5-GFP systems, have become indispensable tools in preclinical research. These systems achieve detection by stably integrating modified green fluorescent protein (GFP) fragments into the host genome. In both detection systems, the GFP gene is initially in an inactive state because its coding sequence contains a rare recognition site for the endonuclease I-SceI [156].

The DR-GFP reporting system uses two tandemly arranged mutant GFP sequences to detect HRR. After I-SceI induces DSBs, only when the cells successfully initiate HRR will a distal non-broken but truncated GFP sequence be used as a template to reconstitute a functional complete GFP gene. At this point, quantitative detection of GFP fluorescence intensity through FACS can provide direct and highly specific functional readings of the cells’ homologous recombination ability [10].

The EJ5-GFP system is used to detect NHEJ. In this system, the promoter is separated from the GFP coding sequence by a filling fragment with I-SceI sites at both ends. After I-SceI digestion removes this filling fragment, if the cells successfully repair the break through NHEJ, the promoter and the GFP gene can reconnect, allowing the expression of the fluorescent protein to resume. Such reporter systems have been instrumental in revealing how post-translational modifications, like the SUMOylation of Ku80, orchestrate NHEJ efficacy and confer radioresistance [32].

Compared to static biochemical markers, these reporter systems can achieve quantitative, dynamic, and high-throughput assessment of specific repair activities in living cells and thus have high application value in gene screening and the preclinical evaluation of novel DDR inhibitors. However, the instability of exogenous I-SceI expression efficiency and the objective requirement that the reporter systems usually need to construct stable cell lines have to some extent limited their direct application in primary patient tissue samples.

To clarify the research tools for each pathway, we summarize the corresponding relationship between key DDR pathways and their targeted detection methods in Table 5.

## 5. Discussion

### 5.1. Development and Status of DDR Detection Techniques

**Figure 3:**
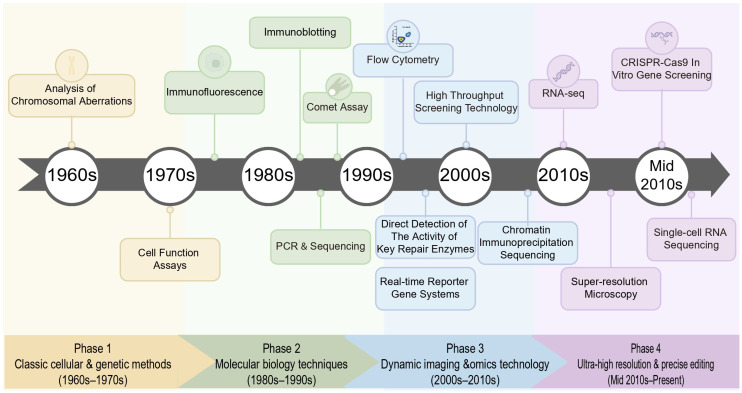
Timeline of the development of different DNA damage assays. Timeline of the development of DNA damage testing technologies.

Figure 3 illustrates the development history of DNA damage detection technologies. This evolution goes beyond the mere accumulation of new tools; it signifies a significant advancement in terms of detection sensitivity, specificity, and temporal resolution. As a result, the DDR is no longer regarded as a passive cellular defense mechanism but is redefined as a highly dynamic, targetable, and significantly spatially and temporally heterogeneous signaling network. The current research paradigm has shifted from simply quantifying the degree of damage to integrating multi-omics analysis and nanoscale imaging techniques to precisely analyze the assembly dynamics of specific repair complexes in specific cell subpopulations and specific genomic loci. This multi-dimensional interpretation of the genome maintenance mechanism has laid a crucial theoretical foundation for modern precision oncology.

### 5.2. Persistent Challenges and Technical Limitations

Although Section 4 elaborates on numerous technological breakthroughs, there are still deep limitations in the research of DDR, namely the disconnection between biochemical damage perception and functional phenotypic outcomes. The challenges in this field do not stem from the mechanism level of a single detection technology but rather from a broader “spatiotemporal compromise”. Currently, experimental design often forces researchers to make a trade-off between capturing the spatial structural changes of the entire genome at high resolution and tracking real-time molecular dynamics (the latter often requires the introduction of exogenous markers that may alter the natural physiological functions of the cells). Moreover, as research shifts from simplified in vitro models to complex tissue microenvironments, how to strictly distinguish primary direct DNA repair activation from secondary pleiotropic stress responses (such as metabolic reprogramming or early apoptosis) remains a core challenge. To address these systemic limitations, we cannot merely stop at optimizing a single detection method.

### 5.3. Future Directions and Integrative Strategies

To overcome the technical limitations of the DDR detection method and promote innovative applications, the future development direction of DDR detection needs to shift towards non-invasive detection methods. The biomarkers of the DDR pathway have significant prognostic value in predicting the response of various cancers to treatment (including sensitivity and resistance) [166], especially by leveraging synthetic lethality (for example, using PARP inhibitors in tumors with homologous recombination deficiency) [167]. However, converting laboratory findings into practical clinical utility for patients requires the development of powerful liquid biopsy technologies [168], for instance, using ctDNA fragmentomics and miRNA analysis related to DDR to monitor the tumor DNA repair dynamics in real time and non-invasively during treatment [169]. These technologies can track the changes in DDR activity in cancer patients in real time and contribute to early diagnosis and efficacy assessment. At the same time, overcoming the existing limitations is likely to require the integration of computational models with experimental data rather than simple biochemical innovations. Multimodal techniques can reveal unknown regulatory networks and discover new therapeutic targets by establishing functional associations between somatic mutations in DNA-damaged cells, post-translational modifications of proteins, and metabolic reprogramming [170]. Moreover, machine learning and deep learning are transforming DDR data analysis models. Automated image processing algorithms can efficiently quantify large amounts of microscopic image data, such as the aggregation of γ-H2AX subunits [171,172,173], and the integration of predictive integration of multimodal features helps discover new biomarkers and improve compound screening efficiency [174]. This AI-driven integration can identify hidden regulatory nodes and predict treatment resistance before clinical symptoms appear, clarifying the technical limitations and clinical translational feasibility of the method [175]. By integrating advanced detection technologies, HTS, and AI analysis methods through interdisciplinary collaboration efforts among experts in molecular biology, computational analysis, and clinical oncology, the discovery of DDR mechanisms will ultimately be transformed into quantifiable, patient-survival-improving precision treatment plans.

## 6. Conclusions and Future Perspectives

As summarized in this review, the progress from traditional chromosome aberration analysis to the current high-throughput, multi-dimensional genomic tools marks a qualitative leap in our ability to detect and analyze DDR. This transformation (resulting from a deeper understanding of the biological nature of DDR: that DDR is not an isolated intracellular mechanism but a complex regulatory network that profoundly affects cancer occurrence and development) has been powerfully confirmed by the clinical success of PARP inhibitors in homologous recombination deficiency-type cancers, indicating that targeting specific DDR defects can lead to significant therapeutic effects [6]. Therefore, DDR detection has risen to become an important technical field that has a decisive impact on cancer diagnosis, prognosis assessment, and patient treatment stratification.

However, the current detection techniques still face numerous challenges; for example, most methods provide static signals, making it difficult to capture the dynamic processes and cellular heterogeneity of DDR; cutting-edge technologies such as super-resolution microscopy and single-cell sequencing are limited by cost, throughput, and operational complexity, hindering their widespread clinical adoption; furthermore, how to translate the abundant DDR information from laboratory research into clinical detection indicators remains a bottleneck that needs to be overcome.

While much remains to be done in the coming years to improve patient outcomes and develop viable detection methods for cancer and other diseases, the convergence of single-molecule detection sensitivity, multi-omics data integration (including spatial transcriptomics), and AI-powered predictive modeling holds the potential to once again reshape the methodological landscape, offering unparalleled insights into oncology, ageing biology, and the fundamental maintenance of genomic integrity.

## Figures and Tables

**Table 2:** Different strategies for assessing DDR—assay performance and practical logistics.

Method	Sensitivity	Specificity	Resolution	Cost (Relative)	Runtime	Throughput	Reference
Analysis of Chromosomal Aberrations	Moderate	High	Cytogenetic level	Moderate	Days	Low	[143]
Micronucleus test	Moderate	High	Cytogenetic level	Moderate	Days	Low	[144]
IF	High	High	Cellular level	Moderate	1–3 days	Low	[145]
Immunoblotting	Moderate	High	Protein level	Low	1–3 days	Low	[146]
PCR and Sequencing	Very High	High	Gene level	Low	1–2 days	Moderate	[147]
Comet assay	High	Moderate	Single-cell DNA strand level	Low	Hours	Moderate	[148]
ChIP-seq	Very High	High	Genome-wide, protein-DNA interaction sites	High	Days	Moderate	[149]
HTS	High	Variable	Population level	Very High	Days–weeks	Very High	[150]
RNA-seq and scRNA-seq	High	High	Transcriptome-wide	High	Weeks	Very High	[151]
CRISPR-Cas9	Moderate	Moderate	Gene level	Very High	Months	High	[152]
Cell Function Tests	Moderate	Moderate	Cellular level	Low	Days–weeks	Low	[153]
FACS	Moderate	High	Cellular level	Moderate	1–2 days	High	[154]
Cell Viability Assay	Moderate	Moderate	Cellular level	Low	Hours–1 day	High	[155]
Real-time Reporter Gene Systems	High	High	Gene or pathway level (live-cell)	Moderate	Hours–days	Moderate	[156]
Super-resolution Microscopy	Very High	Very High	Nanoscale	Very High	Hours-days	Low	[157]
Direct Detection of The Activity of Key Repair Enzymes	Very High	High	Enzymatic activity level	Moderate	Hours–1 day	Low	[138]

Abbreviation: DDR, DNA damage response; IF, Immunofluorescence; ChIP-seq, Chromatin Immunoprecipitation Sequencing; HTS, High-throughput Screening Technology; scRNA-seq: Single-cell RNA Sequencing; FACS: Flow Cytometry.

**Table 3:** Different strategies for assessing DDR—considerations for applicability and standardization.

Method	Replicability	Standardization Efforts	Experimental Pitfalls	Applicable Scenarios	Input Requirements	Typical Controls	Reference
Analysis of Chromosomal Aberrations	High	Well-established	Requires metaphase-arrested cells, time-consuming scoring	Detect large-scale DNA damage or repair deficiency	Actively dividing cells (metaphase spreads)	Normal karyotype sample	[143]
Micronucleus test	High	Improve and establish	Requires active dividing cells, time-consuming scoring, may be insensitive to indirect acting genotoxic substances	Detect large-scale DNA damage or repair defects; follow the standard tests as per current regulatory guidelines	Active dividing cells (mid-stage smear)	Normal karyotype sample; Positive control for genotoxic substances	[40]
IF	Moderate	Partial	Subjectivity in analysis, photobleaching	Localization of DDR markers	Cell quantity/density	Positive control: high-expression cell lines	[145]
Immunoblotting	Moderate	Well-established	Cross-reactivity, quantification issues	Detection of key DDR proteins	Total protein mass	β-Actin as internal reference	[146]
PCR and Sequencing	High	Well-standardized	Primer specificity, RNA quality	DDR-related gene expression	Cell count, RNA/cDNA volume	No template control (nuclease-free water)	[147]
Comet assay	High	Widely used, semi-standardized	Subjective scoring, sensitive to electrophoresis conditions	Detect DNA strand breaks, repair kinetics	Low cell number (10^4^–10^5^)	Untreated cells, positive DNA damage control	[148]
ChIP-seq	High	Ongoing efforts for standardization	Antibody specificity, cross-linking variability	Mapping repair factor	Large cell number, high-quality chromatin	Input DNA and IgG control	[149]
HTS	High	Active standardization in progress	Off-targets, data normalization artifacts	Identifying genes or compounds influencing DNA repair	Cell libraries, genome-scale reagents	Non-targeting control	[150]
RNA-seq and scRNA-seq	High	Emerging standards	Batch effects, library preparation bias	DDR gene expression profiling	Total RNA content or cell count	Biological replicates	[151]
CRISPR-Cas9	Moderate	Limited	Off-target effects, cell line dependency	Screening DDR key genes	Cell count, DNA library size	Internal gRNA library controls	[152]
Cell Function Tests	High	Long-established	Sensitive to culture conditions, subjective outcome scoring	Assess cell proliferation, apoptosis	Cell cultures	Untreated or mock-treated cells	[153]
FACS	High	Well-standardized	Staining variability, gating subjectivity	Quantification of γ-H2AX foci	Cell count	Unstained control	[154]
Cell Viability Assay	High	Highly standardized	Metabolic interference, reagent-dependent variability	Evaluate cellular survival after DNA damage	Cell culture (multi-well plates)	Vehicle-treated controls	[155]
Real-time Reporter Gene Systems	Moderate	Standardized for specific reporters	Reporter leakiness, transfection/transduction efficiency	Monitor dynamic DDR pathway activation	Stably integrated cell lines	Empty vector or baseline reporter	[156]
Super-resolution Microscopy	Low	Limited	Sample preparation complexity, artifacts	Subnuclear DDR protein localization	Cell density	Untreated control	[157]
Direct Detection of The Activity of Key Repair Enzymes	Moderate	Limited standardization	Requires purified enzyme or optimized substrate	Biochemical measurement of specific repair enzyme activity	Cell lysate or purified repair enzyme	Inactive enzyme or buffer-only control	[138]

Abbreviation: DDR, DNA damage response; IF, Immunofluorescence; ChIP-seq, Chromatin Immunoprecipitation Sequencing; HTS, High-throughput Screening Technology; scRNA-seq: Single-cell RNA Sequencing; FACS: Flow Cytometry.

**Table 4:** Different strategies for assessing DDR—specificity and generality of readouts.

Method	DDR or DDR Consequence	Specificity to Damage Type	Generality of Readout	Reference
Analysis of Chromosomal Aberrations	Consequence	Low (General chromosomal damage)	High (Final outcome of various breaks/rearrangements)	[143]
Micronucleus test	Consequence	Low (Product of chromosome breakage or loss)	High (Detects loss of chromosomal integrity)	[144]
IF	DDR	Very High (Localization of specific proteins or modifications)	Low (Targets specific markers)	[145]
Immunoblotting	DDR	High (Abundance of specific proteins or modifications)	Low (Targets specific markers)	[146]
PCR and Sequencing	Consequence	Very High (Specific mutations or repair events)	Low (Targets specific sequences)	[147]
Comet assay	DDR	Medium (Can differentiate single/DSBs)	High (Overall DNA damage)	[148]
ChIP-seq	DDR	Very High (Genomic loci bound by specific proteins)	Medium-Low (Targets specific protein of interest)	[149]
HTS	DDR	Depends on the core assay	Very High (Large-scale parallel screening)	[150]
RNA-seq and scRNA-seq	DDR	Medium (Transcriptional signature of specific pathway activation)	Very High (Genome-wide transcriptional changes)	[151]
CRISPR-Cas9	Tool	High (Dynamics of specific pathway activity)	Low (Targets specific pathways)	[152]
Cell Function Tests	Consequence	Low (Integrated functional output)	High (Long-term outcomes like clonogenic survival)	[153]
FACS	Consequence	Medium-High (Can combine multiple parameters)	High (General phenotypes like cell cycle, apoptosis)	[154]
Cell Viability Assay	Consequence	Low (Final outcome of cell death)	High (General indicator of cellular health status)	[155]
Real-time Reporter Gene Systems	DDR	High (Dynamics of specific pathway activity)	Low (Targets specific pathways)	[156]
Super-resolution Microscopy	DDR	Extremely High (Nanometer-scale localization)	Medium (Capable of multi-target imaging, but field of view is relatively small)	[157]
Direct Detection of The Activity of Key Repair Enzymes	DDR	Very High (Biochemical function of a specific enzyme)	Low (Targets specific enzymes)	[138]

Abbreviation: DDR, DNA damage response; IF, Immunofluorescence; DSBs, double-strand breaks; ChIP-seq, Chromatin Immunoprecipitation Sequencing; HTS, High-throughput Screening Technology; scRNA-seq: Single-cell RNA Sequencing; FACS: Flow Cytometry.

**Table 5:** Correlating Specific DNA Repair Pathways with Targeted Detection Methodologies.

DNA Repair Pathways	Targeted Detection Methodologies	Biological Readout/ Specific Markers	DNA Repair Pathways
DR	MS-PCR for MGMT, O6-MeG Adduct Quantification	Epigenetic silencing status of MGMT promoter, Direct measurement of unrepaired O6-MeG lesions	[163]
BER	Enzyme-modified Comet Assay (FPG/Endo III) PARylation IF	Quantification of specific oxidative base lesions (e.g., 8-oxoguanine) PARP1 hyperactivation and PAR chain synthesis	[73]
NER	UDS Assay XR-seq	EdU/BrdU incorporation in G1/G2 cells (repair synthesis) Genome-wide mapping of excised damage-containing oligonucleotides	[164]
MMR	MSI PCR Assay MutL/MutS Immunohistochemistry	Shift in microsatellite repeat lengths Loss of MLH1, MSH2, MSH6, or PMS2 protein expression	[165]
HR	DR-GFP Reporter Assay RAD51/BRCA1 IF SCE	Reconstitution of GFP fluorescence Formation of chromatin-bound foci in S/G2 phase High-throughput cytogenetic crossing-over events	[156]
NHEJ	EJ5-GFP Reporter Assay 53BP1/DNA-PKcs IF or Immunoblotting	Reconstitution of GFP via end-ligation 53BP1 nuclear bodies (G1 phase); p-DNA-PKcs (Thr2609)	[156]

Abbreviation: DR, direct repair; MS-PCR, Methylation-Specific PCR; MGMT, methylguanine methyltransferase; O6-MeG, O6-methylguanine; BER, base excision repair; IF, Immunofluorescence; NER, nucleotide excision repair; UDS, Unscheduled DNA Synthesis; XR-seq, eXcision Repair Sequencing; MMR, mismatch repair; MSI, Microsatellite Instability; MutL, MLH1PMS2; MutS, MSH2MSH6; HR, homologous recombination; SCE, Sister Chromatid Exchange; NHEJ, nonhomologous end joining.

## Data Availability

No new data were created or analyzed in this study. Data sharing is not applicable to this article.

## References

[1] Jackson S.P., Bartek J. (2009). The DNA-damage response in human biology and disease. Nature.

[2] Abd Al-Razaq M.A., Isermann A., Hecht M., Rübe C.E. (2023). Automated Image Analysis of Transmission Electron Micrographs: Nanoscale Evaluation of Radiation-Induced DNA Damage in the Context of Chromatin. Cells.

[3] Mota M.B.S., Carvalho M.A., Monteiro A.N.A., Mesquita R.D. (2019). DNA damage response and repair in perspective: Aedes aegypti, Drosophila melanogaster and Homo sapiens. Parasit. Vectors.

[4] Qing X., Zhang G., Wang Z.Q. (2023). DNA damage response in neurodevelopment and neuromaintenance. FEBS J..

[5] Yu W., Xu H., Sun Z., Du Y., Sun S., Abudureyimu M., Zhang M., Tao J., Ge J., Ren J. (2023). TBC1D15 deficiency protects against doxorubicin cardiotoxicity via inhibiting DNA-PKcs cytosolic retention and DNA damage. Acta Pharm. Sin. B.

[6] Baxter J.S., Zatreanu D., Pettitt S.J., Lord C.J. (2022). Resistance to DNA repair inhibitors in cancer. Mol. Oncol..

[7] Fu J., Liao L., Balaji K.S., Wei C., Kim J., Peng J. (2021). Epigenetic modification and a role for the E3 ligase RNF40 in cancer development and metastasis. Oncogene.

[8] Tirman S., Cybulla E., Quinet A., Meroni A., Vindigni A. (2021). PRIMPOL ready, set, reprime!. Crit. Rev. Biochem. Mol. Biol..

[9] Sharma D., Singh A., Pathak M., Kaur L., Kumar V., Roy B.G., Ojha H. (2020). DNA binding and antiradical potential of ethyl pyruvate: Key to the DNA radioprotection. Chem. Biol. Interact..

[10] Li J., Sun H., Huang Y., Wang Y., Liu Y., Chen X. (2019). Pathways and assays for DNA double-strand break repair by homologous recombination. Acta Biochim. Biophys. Sin..

[11] Li F., Liu W.C., Wang Q., Sun Y., Wang H., Jin X. (2020). NG2-glia cell proliferation and differentiation by glial growth factor 2 (GGF2), a strategy to promote functional recovery after ischemic stroke. Biochem. Pharmacol..

[12] Moosavi F., Hassani B., Nazari S., Saso L., Firuzi O. (2024). Targeting DNA damage response in pancreatic ductal adenocarcinoma: A review of preclinical and clinical evidence. Biochim. Biophys. Acta Rev. Cancer.

[13] Sharma R., Mishra A., Bhardwaj M., Singh G., Indira Harahap L.V., Vanjani S., Pan C.H., Nepali K. (2025). Medicinal chemistry breakthroughs on ATM, ATR, and DNA-PK inhibitors as prospective cancer therapeutics. J. Enzyme Inhib. Med. Chem..

[14] Matthews H.K., Bertoli C., de Bruin R.A.M. (2022). Cell cycle control in cancer. Nat. Rev. Mol. Cell Biol..

[15] Zhao B., Rothenberg E., Ramsden D.A., Lieber M.R. (2020). The molecular basis and disease relevance of non-homologous DNA end joining. Nat. Rev. Mol. Cell Biol..

[16] He E.Y., Hawkins N.J., Mak G., Roncolato F., Goldstein D., Liauw W., Clingan P., Chin M., Ward R.L. (2016). The Impact of Mismatch Repair Status in Colorectal Cancer on the Decision to Treat with Adjuvant Chemotherapy: An Australian Population-Based Multicenter Study. Oncologist.

[17] El-Hajjar M., Gerhardt L., Hong M.M.Y., Krishnamoorthy M., Figueredo R., Zheng X., Koropatnick J., Maleki Vareki S. (2023). Inducing mismatch repair deficiency sensitizes immune-cold neuroblastoma to anti-CTLA4 and generates broad anti-tumor immune memory. Mol. Ther..

[18] Feng Y., Li C., Stewart J.A., Barbulescu P., Seija Desivo N., Álvarez-Quilón A., Pezo R.C., Perera M.L.W., Chan K., Tong A.H.Y. (2021). FAM72A antagonizes UNG2 to promote mutagenic repair during antibody maturation. Nature.

[19] Solta A., Boettiger K., Kovács I., Lang C., Megyesfalvi Z., Ferk F., Mišík M., Hoetzenecker K., Aigner C., Kowol C.R. (2023). Entinostat Enhances the Efficacy of Chemotherapy in Small Cell Lung Cancer Through S-phase Arrest and Decreased Base Excision Repair. Clin. Cancer Res..

[20] Ray Chaudhuri A., Nussenzweig A. (2017). The multifaceted roles of PARP1 in DNA repair and chromatin remodelling. Nat. Rev. Mol. Cell Biol..

[21] Räz M.H., Aloisi C.M.N., Gahlon H.L., Sturla S.J. (2019). DNA Adduct-Directed Synthetic Nucleosides. Acc. Chem. Res..

[22] Vechtomova Y.L., Telegina T.A., Buglak A.A., Kritsky M.S. (2021). UV Radiation in DNA Damage and Repair Involving DNA-Photolyases and Cryptochromes. Biomedicines.

[23] Liu M., Zhang J., Zhu C., Zhang X., Xiao W., Yan Y., Liu L., Zeng H., Gao Y.Q., Yi C. (2021). DNA repair glycosylase hNEIL1 triages damaged bases via competing interaction modes. Nat. Commun..

[24] Du Y., Zhou Y., Yan X., Pan F., He L., Guo Z., Hu Z. (2024). APE1 inhibition enhances ferroptotic cell death and contributes to hepatocellular carcinoma therapy. Cell Death Differ..

[25] Kuschal C., Thoms K.M., Boeckmann L., Laspe P., Apel A., Schön M.P., Emmert S. (2011). Cyclosporin A inhibits nucleotide excision repair via downregulation of the xeroderma pigmentosum group A and G proteins, which is mediated by calcineurin inhibition. Exp. Dermatol..

[26] Kim J., Li C.L., Chen X., Cui Y., Golebiowski F.M., Wang H., Hanaoka F., Sugasawa K., Yang W. (2023). Lesion recognition by XPC, TFIIH and XPA in DNA excision repair. Nature.

[27] Yu J., Yan C., Paul T., Brewer L., Tsutakawa S.E., Tsai C.L., Hamdan S.M., Tainer J.A., Ivanov I. (2024). Molecular architecture and functional dynamics of the pre-incision complex in nucleotide excision repair. Nat. Commun..

[28] Mengoli V., Ceppi I., Sanchez A., Cannavo E., Halder S., Scaglione S., Gaillard P.H., McHugh P.J., Riesen N., Pettazzoni P. (2023). WRN helicase and mismatch repair complexes independently and synergistically disrupt cruciform DNA structures. Embo J..

[29] Guan J., Lu C., Jin Q., Lu H., Chen X., Tian L., Zhang Y., Ortega J., Zhang J., Siteni S. (2021). MLH1 Deficiency-Triggered DNA Hyperexcision by Exonuclease 1 Activates the cGAS-STING Pathway. Cancer Cell.

[30] Li J., Song C., Gu J., Li C., Zang W., Shi L., Chen L., Zhu L., Zhou M., Wang T. (2023). RBBP4 regulates the expression of the Mre11-Rad50-NBS1 (MRN) complex and promotes DNA double-strand break repair to mediate glioblastoma chemoradiotherapy resistance. Cancer Lett..

[31] Shu Y., Jin X., Ji M., Zhang Z., Wang X., Liang H., Lu S., Dong S., Lin Y., Guo Y. (2024). Ku70 Binding to YAP Alters PARP1 Ubiquitination to Regulate Genome Stability and Tumorigenesis. Cancer Res..

[32] Zhao H., Gao S., Han Y., Xie D., Xuan L., Huang X., Luo J., Ran Q., Li G., Guo H. (2025). Conversion of Ku80 K568 crotonylation to SUMOylation facilitates DNA non-homologous end joining and cancer radioresistance. Signal Transduct. Target. Ther..

[33] Selvaraj S., Feist W.N., Viel S., Vaidyanathan S., Dudek A.M., Gastou M., Rockwood S.J., Ekman F.K., Oseghale A.R., Xu L. (2024). High-efficiency transgene integration by homology-directed repair in human primary cells using DNA-PKcs inhibition. Nat. Biotechnol..

[34] Gupta N., Huang T.T., Horibata S., Lee J.M. (2022). Cell cycle checkpoints and beyond: Exploiting the ATR/CHK1/WEE1 pathway for the treatment of PARP inhibitor-resistant cancer. Pharmacol. Res..

[35] Huang R., Zhou P.K. (2021). DNA damage repair: Historical perspectives, mechanistic pathways and clinical translation for targeted cancer therapy. Signal Transduct. Target. Ther..

[36] Tabrizi S.J., Flower M.D., Ross C.A., Wild E.J. (2020). Huntington disease: New insights into molecular pathogenesis and therapeutic opportunities. Nat. Rev. Neurol..

[37] Yang F., Zhou H., Luo P., Jia L., Hou M., Huang J., Gao L., Zhang Q., Guan Y., Bao H. (2024). Celastrol induces DNA damage and cell death in BCR-ABL T315I-mutant CML by targeting YY1 and HMCES. Phytomedicine.

[38] Jin C., Tao X., Zhang W., Xu H., Wu Y., Chen Q., Li S., Ning A., Wang W., Wu Q. (2024). Multi-omics and multi-stages integration identified a novel variant associated with silicosis risk. Arch. Toxicol..

[39] Ahluwalia K.K., Thakur K., Ahluwalia A.S., Hashem A., Avila-Quezada G.D., Abd Allah E.F., Thakur N. (2023). Assessment of Genotoxicity of Zinc Oxide Nanoparticles Using Mosquito as Test Model. Toxics.

[40] Thybaud V., Lorge E., Levy D.D., van Benthem J., Douglas G.R., Marchetti F., Moore M.M., Schoeny R. (2017). Main issues addressed in the 2014-2015 revisions to the OECD Genetic Toxicology Test Guidelines. Environ. Mol. Mutagen..

[41] Costa M.I., Lapa B.S., Jorge J., Alves R., Carreira I.M., Sarmento-Ribeiro A.B., Gonçalves A.C. (2022). Zinc Prevents DNA Damage in Normal Cells but Shows Genotoxic and Cytotoxic Effects in Acute Myeloid Leukemia Cells. Int. J. Mol. Sci..

[42] Hong X., Liu W., Song R., Shah J.J., Feng X., Tsang C.K., Morgan K.M., Bunting S.F., Inuzuka H., Zheng X.F. (2016). SOX9 is targeted for proteasomal degradation by the E3 ligase FBW7 in response to DNA damage. Nucleic Acids Res..

[43] Afiahayati, Anarossi E., Yanuaryska R.D., Mulyana S. (2022). GamaComet: A Deep Learning-Based Tool for the Detection and Classification of DNA Damage from Buccal Mucosa Comet Assay Images. Diagnostics.

[44] Shahsavari F., Mikaeli S., Ghorbanpour M. (2022). Micronucleus assay in the exfoliated cells of buccal mucosa of gasoline station workers in Tehran. J. Cancer Res. Ther..

[45] Fischer B.C., Musengi Y., Herrmann K., Kneuer C., König J. (2026). How the methodology determines the outcome of the in vitro micronucleus assay (OECD TG 487): A comparison of the MicroFlow and the microscopic evaluation approach highlights the impact of cytotoxicity/cytostasis metrics in V79 cells for matrine. Arch. Toxicol..

[46] Bernacki D.T., Bryce S.M., Bemis J.C., Dertinger S.D., Witt K.L., Smith-Roe S.L. (2019). Evidence for an Aneugenic Mechanism of Action for Micronucleus Induction by Black Cohosh Extract. Environ. Mol. Mutagen..

[47] Girardi L., Figliuzzi M., Poli M., Serdarogullari M., Patassini C., Caroselli S., Pergher I., Cogo F., Coban O., Boynukalin F.K. (2023). The use of copy number loads to designate mosaicism in blastocyst stage PGT-A cycles: Fewer is better. Hum. Reprod..

[48] Cao X., Sun Y., Lu P., Zhao M. (2020). Fluorescence imaging of intracellular nucleases-A review. Anal. Chim. Acta.

[49] Lopez Chiloeches M., Bergonzini A., Martin O.C.B., Bergstein N., Erttmann S.F., Aung K.M., Gekara N.O., Avila Cariño J.F., Pateras I.S., Frisan T. (2023). Genotoxin-producing Salmonella enterica induces tissue-specific types of DNA damage and DNA damage response outcomes. Front. Immunol..

[50] Liu Y., Zou R.S., He S., Nihongaki Y., Li X., Razavi S., Wu B., Ha T. (2020). Very fast CRISPR on demand. Science.

[51] Sebastian R., Sun E.G., Fedkenheuer M., Fu H., Jung S., Thakur B.L., Redon C.E., Pegoraro G., Tran A.D., Gross J.M. (2025). Mechanism for local attenuation of DNA replication at double-strand breaks. Nature.

[52] Huo D., Chen H., Cheng Y., Song X., Zhang K., Li M.J., Xuan C. (2020). JMJD6 modulates DNA damage response through downregulating H4K16ac independently of its enzymatic activity. Cell Death Differ..

[53] Dibitetto D., Liptay M., Vivalda F., Dogan H., Gogola E., González Fernández M., Duarte A., Schmid J.A., Decollogny M., Francica P. (2024). H2AX promotes replication fork degradation and chemosensitivity in BRCA-deficient tumours. Nat. Commun..

[54] Li Q., Zhang P., Hu H., Huang H., Pan D., Mao G., Hu B. (2022). The DDR-related gene signature with cell cycle checkpoint function predicts prognosis, immune activity, and chemoradiotherapy response in lung adenocarcinoma. Respir. Res..

[55] Cardano M., Buscemi G., Zannini L. (2022). Sex disparities in DNA damage response pathways: Novel determinants in cancer formation and therapy. iScience.

[56] Yang J., Qi L., Chiang H.C., Yuan B., Li R., Hu Y. (2021). BRCA1 Antibodies Matter. Int. J. Biol. Sci..

[57] Lehle S., Hildebrand D.G., Merz B., Malak P.N., Becker M.S., Schmezer P., Essmann F., Schulze-Osthoff K., Rothfuss O. (2014). LORD-Q: A long-run real-time PCR-based DNA-damage quantification method for nuclear and mitochondrial genome analysis. Nucleic Acids Res..

[58] Kuang X., Zhao W., Wang Q., Sun Z., Xu F., Geng R., Li B., Zheng T., Zheng Q. (2024). RNA-seq analysis highlights DNA replication and DNA repair associated with early-onset hearing loss in the cochlea of DBA/2J mice. Life Sci..

[59] Li Z., Zhang W., Li S., Tao X., Xu H., Wu Y., Chen Q., Ning A., Tian T., Zhang L. (2024). Integration of apaQTL and eQTL analysis reveals novel SNPs associated with occupational pulmonary fibrosis risk. Arch. Toxicol..

[60] Luchini C., Bibeau F., Ligtenberg M.J.L., Singh N., Nottegar A., Bosse T., Miller R., Riaz N., Douillard J.Y., Andre F. (2019). ESMO recommendations on microsatellite instability testing for immunotherapy in cancer, and its relationship with PD-1/PD-L1 expression and tumour mutational burden: A systematic review-based approach. Ann. Oncol..

[61] Furda A., Santos J.H., Meyer J.N., Van Houten B. (2014). Quantitative PCR-based measurement of nuclear and mitochondrial DNA damage and repair in mammalian cells. Methods Mol. Biol..

[62] Lin Q., Zeng R., Yang J., Xu Z., Jin S., Wei G. (2024). Prognostic stratification of sepsis through DNA damage response based RiskScore system: Insights from single-cell RNA-sequencing and transcriptomic profiling. Front. Immunol..

[63] Habas K., Najafzadeh M., Baumgartner A., Brinkworth M.H., Anderson D. (2017). An evaluation of DNA damage in human lymphocytes and sperm exposed to methyl methanesulfonate involving the regulation pathways associated with apoptosis. Chemosphere.

[64] Shioi T., Hatazawa S., Oya E., Hosoya N., Kobayashi W., Ogasawara M., Kobayashi T., Takizawa Y., Kurumizaka H. (2024). Cryo-EM structures of RAD51 assembled on nucleosomes containing a DSB site. Nature.

[65] Zhang J., Hu W., Liu K., Liu J., Zheng Y., Sun X., Mei L., Qian Z., Sun Q., Liu Q. (2023). Integrated mRNA and microRNA profiling in lung tissue and blood from human silicosis. J. Gene Med..

[66] Debiais M., Lelievre A., Smietana M., Müller S. (2020). Splitting aptamers and nucleic acid enzymes for the development of advanced biosensors. Nucleic Acids Res..

[67] Smitalova D., Dvorakova D., Racil Z., Romzova M. (2021). Digital PCR can provide improved BCR-ABL1 detection in chronic myeloid leukemia patients in deep molecular response and sensitivity of standard quantitative methods using EAC assays. Pract. Lab. Med..

[68] Wales S.Q., Pandiscia A., Kulka M., Sanchez G., Randazzo W. (2024). Challenges for estimating human norovirus infectivity by viability RT-qPCR as compared to replication in human intestinal enteroids. Int. J. Food Microbiol..

[69] Zhang J., Sun J., Gu X., Shen Y., Sun H. (2024). Transcriptome sequencing analysis reveals the molecular regulatory mechanism of myocardial hypertrophy induced by angiotensin II. Biochem. Pharmacol..

[70] de Lapuente J., Lourenço J., Mendo S.A., Borràs M., Martins M.G., Costa P.M., Pacheco M. (2015). The Comet Assay and its applications in the field of ecotoxicology: A mature tool that continues to expand its perspectives. Front. Genet..

[71] Copp M.E., Chubinskaya S., Bracey D.N., Shine J., Sessions G., Loeser R.F., Diekman B.O. (2022). Comet assay for quantification of the increased DNA damage burden in primary human chondrocytes with aging and osteoarthritis. Aging Cell.

[72] Milić M., Ceppi M., Bruzzone M., Azqueta A., Brunborg G., Godschalk R., Koppen G., Langie S., Møller P., Teixeira J.P. (2021). The hCOMET project: International database comparison of results with the comet assay in human biomonitoring. Baseline frequency of DNA damage and effect of main confounders. Mutat. Res. Rev. Mutat. Res..

[73] Collins A., Møller P., Gajski G., Vodenková S., Abdulwahed A., Anderson D., Bankoglu E.E., Bonassi S., Boutet-Robinet E., Brunborg G. (2023). Measuring DNA modifications with the comet assay: A compendium of protocols. Nat. Protoc..

[74] McKelvey-Martin V.J., Green M.H., Schmezer P., Pool-Zobel B.L., De Méo M.P., Collins A. (1993). The single cell gel electrophoresis assay (comet assay): A European review. Mutat. Res..

[75] Tice R.R., Agurell E., Anderson D., Burlinson B., Hartmann A., Kobayashi H., Miyamae Y., Rojas E., Ryu J.C., Sasaki Y.F. (2000). Single cell gel/comet assay: Guidelines for in vitro and in vivo genetic toxicology testing. Environ. Mol. Mutagen..

[76] (2002). Comparative analysis of baseline 8-oxo-7,8-dihydroguanine in mammalian cell DNA, by different methods in different laboratories: An approach to consensus. Carcinogenesis.

[77] Sibony-Benyamini H., Jbara R., Shubash Napso T., Abu-Rahmoun L., Vizenblit D., Easton-Mor M., Perez S., Brandis A., Leshem T., Peretz A. (2025). The landscape of Helicobacter pylori-mediated DNA breaks links bacterial genotoxicity to its oncogenic potential. Genome Med..

[78] May S., Hirsch C., Rippl A., Bürkle A., Wick P. (2022). Assessing Genotoxicity of Ten Different Engineered Nanomaterials by the Novel Semi-Automated FADU Assay and the Alkaline Comet Assay. Nanomaterials.

[79] Azqueta A., Ladeira C., Giovannelli L., Boutet-Robinet E., Bonassi S., Neri M., Gajski G., Duthie S., Del Bo C., Riso P. (2020). Application of the comet assay in human biomonitoring: An hCOMET perspective. Mutat. Res. Rev. Mutat. Res..

[80] Liu X., Zhang S., An Y., Xu B., Yan G., Sun M. (2025). USP10/XAB2/ANXA2 axis promotes DNA damage repair to enhance chemoresistance to oxaliplatin in colorectal cancer. J. Exp. Clin. Cancer Res..

[81] Buenrostro J.D., Giresi P.G., Zaba L.C., Chang H.Y., Greenleaf W.J. (2013). Transposition of native chromatin for fast and sensitive epigenomic profiling of open chromatin, DNA-binding proteins and nucleosome position. Nat. Methods.

[82] Kou S., Lu Z., Deng D., Ye M., Sui Y., Qin L., Feng T., Jiang Z., Meng J., Lin C.P. (2025). Activation of Imprinted Gene PW1 Promotes Cardiac Fibrosis After Ischemic Injury. Circulation.

[83] Yang W., Wei C., Chen J., Lin Q., Qin Y., Huang T., Deng X., Li M.J., Tang Z., Fang M. (2025). Clonorchis sinensis infection remodels chromatin accessibility in hepatocellular carcinoma. Parasit. Vectors.

[84] Zhou Q., Chen X., He H., Peng S., Zhang Y., Zhang J., Cheng L., Liu S., Huang M., Xie R. (2021). WD repeat domain 5 promotes chemoresistance and Programmed Death-Ligand 1 expression in prostate cancer. Theranostics.

[85] Lareau C.A., Liu V., Muus C., Praktiknjo S.D., Nitsch L., Kautz P., Sandor K., Yin Y., Gutierrez J.C., Pelka K. (2023). Mitochondrial single-cell ATAC-seq for high-throughput multi-omic detection of mitochondrial genotypes and chromatin accessibility. Nat. Protoc..

[86] Xie S.Y., Liu S.Q., Zhang T., Shi W.K., Xing Y., Fang W.X., Zhang M., Chen M.Y., Xu S.C., Fan M.Q. (2024). USP28 Serves as a Key Suppressor of Mitochondrial Morphofunctional Defects and Cardiac Dysfunction in the Diabetic Heart. Circulation.

[87] Lu Z., Wang Z., Song Z., Chen C., Ma H., Gong P., Xu Y. (2022). Single-cell sequencing of brain tissues reveal the central nervous system’s susceptibility to SARS-CoV-2 and the drug. Front. Pharmacol..

[88] Alvarez S., da Silva Almeida A.C., Albero R., Biswas M., Barreto-Galvez A., Gunning T.S., Shaikh A., Aparicio T., Wendorff A., Piovan E. (2022). Functional mapping of PHF6 complexes in chromatin remodeling, replication dynamics, and DNA repair. Blood.

[89] Wu M., Tang W., Chen Y., Xue L., Dai J., Li Y., Zhu X., Wu C., Xiong J., Zhang J. (2024). Spatiotemporal transcriptomic changes of human ovarian aging and the regulatory role of FOXP1. Nat. Aging.

[90] Yimit A., Adebali O., Sancar A., Jiang Y. (2019). Differential damage and repair of DNA-adducts induced by anti-cancer drug cisplatin across mouse organs. Nat. Commun..

[91] Replogle J.M., Norman T.M., Xu A., Hussmann J.A., Chen J., Cogan J.Z., Meer E.J., Terry J.M., Riordan D.P., Srinivas N. (2020). Combinatorial single-cell CRISPR screens by direct guide RNA capture and targeted sequencing. Nat. Biotechnol..

[92] Hussmann J.A., Ling J., Ravisankar P., Yan J., Cirincione A., Xu A., Simpson D., Yang D., Bothmer A., Cotta-Ramusino C. (2021). Mapping the genetic landscape of DNA double-strand break repair. Cell.

[93] Ngoi N.Y.L., Gallo D., Torrado C., Nardo M., Durocher D., Yap T.A. (2025). Synthetic lethal strategies for the development of cancer therapeutics. Nat. Rev. Clin. Oncol..

[94] Topatana W., Juengpanich S., Li S., Cao J., Hu J., Lee J., Suliyanto K., Ma D., Zhang B., Chen M. (2020). Advances in synthetic lethality for cancer therapy: Cellular mechanism and clinical translation. J. Hematol. Oncol..

[95] Hanna R.E., Hegde M., Fagre C.R., DeWeirdt P.C., Sangree A.K., Szegletes Z., Griffith A., Feeley M.N., Sanson K.R., Baidi Y. (2021). Massively parallel assessment of human variants with base editor screens. Cell.

[96] Zimmermann M., Murina O., Reijns M.A.M., Agathanggelou A., Challis R., Tarnauskaitė Ž., Muir M., Fluteau A., Aregger M., McEwan A. (2018). CRISPR screens identify genomic ribonucleotides as a source of PARP-trapping lesions. Nature.

[97] Condon K.J., Orozco J.M., Adelmann C.H., Spinelli J.B., van der Helm P.W., Roberts J.M., Kunchok T., Sabatini D.M. (2021). Genome-wide CRISPR screens reveal multitiered mechanisms through which mTORC1 senses mitochondrial dysfunction. Proc. Natl. Acad. Sci. USA.

[98] Cuella-Martin R., Hayward S.B., Fan X., Chen X., Huang J.W., Taglialatela A., Leuzzi G., Zhao J., Rabadan R., Lu C. (2021). Functional interrogation of DNA damage response variants with base editing screens. Cell.

[99] Wang C., Wang G., Feng X., Shepherd P., Zhang J., Tang M., Chen Z., Srivastava M., McLaughlin M.E., Navone N.M. (2019). Genome-wide CRISPR screens reveal synthetic lethality of RNASEH2 deficiency and ATR inhibition. Oncogene.

[100] Behan F.M., Iorio F., Picco G., Gonçalves E., Beaver C.M., Migliardi G., Santos R., Rao Y., Sassi F., Pinnelli M. (2019). Prioritization of cancer therapeutic targets using CRISPR-Cas9 screens. Nature.

[101] Pan X., Pei X., Huang H., Su N., Wu Z., Wu Z., Qi X. (2021). One-in-one individual package and delivery of CRISPR/Cas9 ribonucleoprotein using apoferritin. J. Control. Release.

[102] Olivieri M., Cho T., Álvarez-Quilón A., Li K., Schellenberg M.J., Zimmermann M., Hustedt N., Rossi S.E., Adam S., Melo H. (2020). A Genetic Map of the Response to DNA Damage in Human Cells. Cell.

[103] Sato Y., Passerini L., Piening B.D., Uyeda M.J., Goodwin M., Gregori S., Snyder M.P., Bertaina A., Roncarolo M.G., Bacchetta R. (2020). Human-engineered Treg-like cells suppress FOXP3-deficient T cells but preserve adaptive immune responses in vivo. Clin. Transl. Immunol..

[104] Ashrafizadeh M., Zarrabi A., Bigham A., Taheriazam A., Saghari Y., Mirzaei S., Hashemi M., Hushmandi K., Karimi-Maleh H., Nazarzadeh Zare E. (2023). (Nano)platforms in breast cancer therapy: Drug/gene delivery, advanced nanocarriers and immunotherapy. Med. Res. Rev..

[105] Bock C., Datlinger P., Chardon F., Coelho M.A., Dong M.B., Lawson K.A., Lu T., Maroc L., Norman T.M., Song B. (2022). High-content CRISPR screening. Nat. Rev. Methods Primers.

[106] Gao Y., Guitton-Sert L., Dessapt J., Coulombe Y., Rodrigue A., Milano L., Blondeau A., Larsen N.B., Duxin J.P., Hussein S. (2023). A CRISPR-Cas9 screen identifies EXO1 as a formaldehyde resistance gene. Nat. Commun..

[107] Zhao R., Liu Y., Zhang H., Chai C., Wang J., Jiang W., Gu Y. (2019). CRISPR-Cas12a-Mediated Gene Deletion and Regulation in Clostridium ljungdahlii and Its Application in Carbon Flux Redirection in Synthesis Gas Fermentation. ACS Synth. Biol..

[108] Kaufmann T., Herbert S., Hackl B., Besold J.M., Schramek C., Gotzmann J., Elsayad K., Slade D. (2020). Direct measurement of protein-protein interactions by FLIM-FRET at UV laser-induced DNA damage sites in living cells. Nucleic Acids Res..

[109] Gassman N.R., Wilson S.H. (2015). Micro-irradiation tools to visualize base excision repair and single-strand break repair. DNA Repair.

[110] Huang J., Wu C., Kloeber J.A., Gao H., Gao M., Zhu Q., Chang Y., Zhao F., Guo G., Luo K. (2023). SLFN5-mediated chromatin dynamics sculpt higher-order DNA repair topology. Mol. Cell.

[111] Berzsenyi I., Pantazi V., Borsos B.N., Pankotai T. (2021). Systematic overview on the most widespread techniques for inducing and visualizing the DNA double-strand breaks. Mutat. Res. Rev. Mutat. Res..

[112] Sánchez H., Paul M.W., Grosbart M., van Rossum-Fikkert S.E., Lebbink J.H.G., Kanaar R., Houtsmuller A.B., Wyman C. (2017). Architectural plasticity of human BRCA2-RAD51 complexes in DNA break repair. Nucleic Acids Res..

[113] Varga D., Majoros H., Ujfaludi Z., Erdélyi M., Pankotai T. (2019). Quantification of DNA damage induced repair focus formation via super-resolution dSTORM localization microscopy. Nanoscale.

[114] Wu R., Liu W., Sun Y., Shen C., Guo J., Zhao J., Mao G., Li Y., Du G. (2020). Nanoscale insight into chromatin remodeling and DNA repair complex in HeLa cells after ionizing radiation. DNA Repair.

[115] Lopez Perez R., Best G., Nicolay N.H., Greubel C., Rossberger S., Reindl J., Dollinger G., Weber K.J., Cremer C., Huber P.E. (2016). Superresolution light microscopy shows nanostructure of carbon ion radiation-induced DNA double-strand break repair foci. FASEB J..

[116] Fu Q., Wang J., Huang T. (2019). Characterizing the DNA damage response in fibrosarcoma stem cells by in-situ cell tracking. Int. J. Radiat. Biol..

[117] Durdik M., Kosik P., Jakl L., Kozackova M., Markova E., Vigasova K., Beresova K., Jakubikova J., Horvathova E., Zastko L. (2021). Imaging flow cytometry and fluorescence microscopy in assessing radiation response in lymphocytes from umbilical cord blood and cancer patients. Cytom. Part A.

[118] Huang M., Yao F., Nie L., Wang C., Su D., Zhang H., Li S., Tang M., Feng X., Yu B. (2023). FACS-based genome-wide CRISPR screens define key regulators of DNA damage signaling pathways. Mol. Cell.

[119] Valente D., Gentileschi M.P., Valenti A., Burgio M., Soddu S., Bruzzaniti V., Guerrisi A., Verdina A. (2024). Cumulative Dose from Recurrent CT Scans: Exploring the DNA Damage Response in Human Non-Transformed Cells. Int. J. Mol. Sci..

[120] Huang M., Feng X., Su D., Wang G., Wang C., Tang M., Paulucci-Holthauzen A., Hart T., Chen J. (2020). Genome-wide CRISPR screen uncovers a synergistic effect of combining Haspin and Aurora kinase B inhibition. Oncogene.

[121] Gong X., Cheng J., Zhang K., Wang Y., Li S., Luo Y. (2022). Transcriptome sequencing reveals Gastrodia elata Blume could increase the cell viability of eNPCs under hypoxic condition by improving DNA damage repair ability. J. Ethnopharmacol..

[122] Wang H., Zhang X., Teng L., Legerski R.J. (2015). DNA damage checkpoint recovery and cancer development. Exp. Cell Res..

[123] Liu C., Liao K., Gross N., Wang Z., Li G., Zuo W., Zhong S., Zhang Z., Zhang H., Yang J. (2020). Homologous recombination enhances radioresistance in hypopharyngeal cancer cell line by targeting DNA damage response. Oral. Oncol..

[124] Chen Y., Jiang T., Zhang H., Gou X., Han C., Wang J., Chen A.T., Ma J., Liu J., Chen Z. (2020). LRRC31 inhibits DNA repair and sensitizes breast cancer brain metastasis to radiation therapy. Nat. Cell Biol..

[125] Tong Y., Wang F., Li S., Guo W., Li Q., Qian Y., Li L., Zhao H., Zhang Y., Gao W.Q. (2024). Histone methyltransferase KMT5C drives liver cancer progression and directs therapeutic response to PARP inhibitors. Hepatology.

[126] Lu T., Pan Y., Kao S.Y., Li C., Kohane I., Chan J., Yankner B.A. (2004). Gene regulation and DNA damage in the ageing human brain. Nature.

[127] Sun J., Zhu Z., Li W., Shen M., Cao C., Sun Q., Guo Z., Liu L., Wu D. (2020). UBE2T-regulated H2AX monoubiquitination induces hepatocellular carcinoma radioresistance by facilitating CHK1 activation. J. Exp. Clin. Cancer Res..

[128] Molkentine J.M., Molkentine D.P., Bridges K.A., Xie T., Yang L., Sheth A., Heffernan T.P., Clump D.A., Faust A.Z., Ferris R.L. (2021). Targeting DNA damage response in head and neck cancers through abrogation of cell cycle checkpoints. Int. J. Radiat. Biol..

[129] Mei C., Sun Z.E., Tan L.M., Gong J.P., Li X., Liu Z.Q. (2022). eIF3a-PPP2R5A-mediated ATM/ATR dephosphorylation is essential for irinotecan-induced DNA damage response. Cell Prolif..

[130] Mathias B., O’Leary D., Saucier N., Ahmad F., White L.S., Russell L., Shinawi M., Smith M.J., Abraham R.S., Cooper M.A. (2024). MYSM1 attenuates DNA damage signals triggered by physiologic and genotoxic DNA breaks. J. Allergy Clin. Immunol..

[131] Welz L., Kakavand N., Hang X., Laue G., Ito G., Silva M.G., Plattner C., Mishra N., Tengen F., Ogris C. (2022). Epithelial X-Box Binding Protein 1 Coordinates Tumor Protein p53-Driven DNA Damage Responses and Suppression of Intestinal Carcinogenesis. Gastroenterology.

[132] Schwalm M.P., Saxena K., Müller S., Knapp S. (2024). Luciferase- and HaloTag-based reporter assays to measure small-molecule-induced degradation pathway in living cells. Nat. Protoc..

[133] Stewart-Ornstein J., Lahav G. (2017). p53 dynamics in response to DNA damage vary across cell lines and are shaped by efficiency of DNA repair and activity of the kinase ATM. Sci. Signal.

[134] Majumdar C., Demir M., Merrill S.R., Hashemian M., David S.S. (2024). FSHing for DNA Damage: Key Features of MutY Detection of 8-Oxoguanine:Adenine Mismatches. Acc. Chem. Res..

[135] Chen Y., Wu J., Zhai L., Zhang T., Yin H., Gao H., Zhao F., Wang Z., Yang X., Jin M. (2024). Metabolic regulation of homologous recombination repair by MRE11 lactylation. Cell.

[136] da Costa A., Chowdhury D., Shapiro G.I., D’Andrea A.D., Konstantinopoulos P.A. (2023). Targeting replication stress in cancer therapy. Nat. Rev. Drug Discov..

[137] Xin C., Chao Z., Xian W., Zhonggao W., Tao L. (2020). The phosphorylation of CHK1 at Ser345 regulates the phenotypic switching of vascular smooth muscle cells both in vitro and in vivo. Atherosclerosis.

[138] Zhang H., Gao Z., He F., Lan J., Chai H., Zhang S., Zuo X., Chen H., Chen X. (2022). Generation of 3′-OH terminal-triggered encoding of multicolor fluorescence for simultaneous detection of different DNA glycosylases. Anal. Bioanal. Chem..

[139] Wu H., Han B.W., Liu T., Zhang M., Wu Y., Nie J. (2024). Epstein-Barr virus deubiquitinating enzyme BPLF1 is involved in EBV carcinogenesis by affecting cellular genomic stability. Neoplasia.

[140] Zarghami N., Murrell D.H., Jensen M.D., Dick F.A., Chambers A.F., Foster P.J., Wong E. (2018). Half brain irradiation in a murine model of breast cancer brain metastasis: Magnetic resonance imaging and histological assessments of dose-response. Radiat. Oncol..

[141] Hsiao H.W., Yang C.C., Masai H. (2023). Claspin-Dependent and -Independent Chk1 Activation by a Panel of Biological Stresses. Biomolecules.

[142] Wang H., Zhang S., Song L., Qu M., Zou Z. (2020). Synergistic lethality between PARP-trapping and alantolactone-induced oxidative DNA damage in homologous recombination-proficient cancer cells. Oncogene.

[143] Mantere T., Neveling K., Pebrel-Richard C., Benoist M., van der Zande G., Kater-Baats E., Baatout I., van Beek R., Yammine T., Oorsprong M. (2021). Optical genome mapping enables constitutional chromosomal aberration detection. Am. J. Hum. Genet..

[144] Seo J.E., Li X., Le Y., Mei N., Zhou T., Guo X. (2023). High-throughput micronucleus assay using three-dimensional HepaRG spheroids for in vitro genotoxicity testing. Arch. Toxicol..

[145] Kinders R.J., Hollingshead M., Lawrence S., Ji J., Tabb B., Bonner W.M., Pommier Y., Rubinstein L., Evrard Y.A., Parchment R.E. (2010). Development of a validated immunofluorescence assay for γH2AX as a pharmacodynamic marker of topoisomerase I inhibitor activity. Clin. Cancer Res..

[146] Janes K.A. (2015). An analysis of critical factors for quantitative immunoblotting. Sci. Signal.

[147] Su Z., Łabaj P.P., Li S., Thierry-Mieg J., Thierry-Mieg D., Shi W., Wang C., Schroth G.P., Setterquist R.A., Thompson J.F. (2014). A comprehensive assessment of RNA-seq accuracy, reproducibility and information content by the Sequencing Quality Control Consortium. Nat. Biotechnol..

[148] Rosenberger A., Rössler U., Hornhardt S., Sauter W., Bickeböller H., Wichmann H.E., Gomolka M. (2011). Validation of a fully automated COMET assay: 1.75 million single cells measured over a 5 year period. DNA Repair.

[149] Landt S.G., Marinov G.K., Kundaje A., Kheradpour P., Pauli F., Batzoglou S., Bernstein B.E., Bickel P., Brown J.B., Cayting P. (2012). ChIP-seq guidelines and practices of the ENCODE and modENCODE consortia. Genome Res..

[150] Valerie N.C.K., Sanjiv K., Mortusewicz O., Zhang S.M., Alam S., Pires M.J., Stigsdotter H., Rasti A., Langelier M.F., Rehling D. (2024). Coupling cellular drug-target engagement to downstream pharmacology with CeTEAM. Nat. Commun..

[151] Holcomb I., Bansal N., Duong T., Babb P., Laliberte J., Swaminathan K., Xu H.H., Melloy L.A., Hildebrand J., Farmer A. (2021). Benchmarking Single-Cell mRNA-Sequencing Technologies Uncovers Differences in Sensitivity and Reproducibility in Cell Types with Low RNA Content. J. Biomol. Tech..

[152] Awwad S.W., Serrano-Benitez A., Thomas J.C., Gupta V., Jackson S.P. (2023). Revolutionizing DNA repair research and cancer therapy with CRISPR-Cas screens. Nat. Rev. Mol. Cell Biol..

[153] Gomes N.P., Frederick B., Jacobsen J.R., Chapnick D., Su T.T. (2023). A High Throughput Screen with a Clonogenic Endpoint to Identify Radiation Modulators of Cancer. Radiat. Res..

[154] Felgentreff K., Baumann U., Klemann C., Schuetz C., Viemann D., Wetzke M., Pannicke U., von Hardenberg S., Auber B., Debatin K.M. (2022). Biomarkers of DNA Damage Response Enable Flow Cytometry-Based Diagnostic to Identify Inborn DNA Repair Defects in Primary Immunodeficiencies. J. Clin. Immunol..

[155] Ghasemi M., Turnbull T., Sebastian S., Kempson I. (2021). The MTT Assay: Utility, Limitations, Pitfalls, and Interpretation in Bulk and Single-Cell Analysis. Int. J. Mol. Sci..

[156] Rajendra E., Grande D., Mason B., Di Marcantonio D., Armstrong L., Hewitt G., Elinati E., Galbiati A., Boulton S.J., Heald R.A. (2024). Quantitative, titratable and high-throughput reporter assays to measure DNA double strand break repair activity in cells. Nucleic Acids Res..

[157] Morgan S.T.B., Whelan D.R., Rozario A.M. (2024). Visualizing DNA damage and repair using single molecule super resolution microscopy. Methods Cell Biol..

[158] Wilsker D.F., Barrett A.M., Dull A.B., Lawrence S.M., Hollingshead M.G., Chen A., Kummar S., Parchment R.E., Doroshow J.H., Kinders R.J. (2019). Evaluation of Pharmacodynamic Responses to Cancer Therapeutic Agents Using DNA Damage Markers. Clin. Cancer Res..

[159] Paul C.D., Mistriotis P., Konstantopoulos K. (2017). Cancer cell motility: Lessons from migration in confined spaces. Nat. Rev. Cancer.

[160] Lloyd R.L., Urban V., Muñoz-Martínez F., Ayestaran I., Thomas J.C., de Renty C., O’Connor M.J., Forment J.V., Galanty Y., Jackson S.P. (2021). Loss of Cyclin C or CDK8 provides ATR inhibitor resistance by suppressing transcription-associated replication stress. Nucleic Acids Res..

[161] Xie H., Wang W., Qi W., Jin W., Xia B. (2021). Targeting DNA Repair Response Promotes Immunotherapy in Ovarian Cancer: Rationale and Clinical Application. Front. Immunol..

[162] Wojtala M., Dąbek A., Rybaczek D., Śliwińska A., Świderska E., Słapek K., El-Osta A., Balcerczyk A. (2019). Silencing Lysine-Specific Histone Demethylase 1 (LSD1) Causes Increased HP1-Positive Chromatin, Stimulation of DNA Repair Processes, and Dysregulation of Proliferation by Chk1 Phosphorylation in Human Endothelial Cells. Cells.

[163] Macagno M., Pessei V., Congiusta N., Lazzari L., Bellomo S.E., Idrees F., Cavaliere A., Pietrantonio F., Raimondi A., Gusmaroli E. (2024). A Comparative Study of Methyl-BEAMing and Droplet Digital PCR for MGMT Gene Promoter Hypermethylation Detection. Diagnostics.

[164] Hu J., Li W., Adebali O., Yang Y., Oztas O., Selby C.P., Sancar A. (2019). Genome-wide mapping of nucleotide excision repair with XR-seq. Nat. Protoc..

[165] Bartley A.N., Mills A.M., Konnick E., Overman M., Ventura C.B., Souter L., Colasacco C., Stadler Z.K., Kerr S., Howitt B.E. (2022). Mismatch Repair and Microsatellite Instability Testing for Immune Checkpoint Inhibitor Therapy: Guideline from the College of American Pathologists in Collaboration with the Association for Molecular Pathology and Fight Colorectal Cancer. Arch. Pathol. Lab. Med..

[166] Zhu Y., Hu J., Hu Y., Liu W. (2009). Targeting DNA repair pathways: A novel approach to reduce cancer therapeutic resistance. Cancer Treat. Rev..

[167] Padella A., Ghelli Luserna Di Rorà A., Marconi G., Ghetti M., Martinelli G., Simonetti G. (2022). Targeting PARP proteins in acute leukemia: DNA damage response inhibition and therapeutic strategies. J. Hematol. Oncol..

[168] Grypari I.M., Tzelepi V., Gyftopoulos K. (2023). DNA Damage Repair Pathways in Prostate Cancer: A Narrative Review of Molecular Mechanisms, Emerging Biomarkers and Therapeutic Targets in Precision Oncology. Int. J. Mol. Sci..

[169] Santini D., Botticelli A., Galvano A., Iuliani M., Incorvaia L., Gristina V., Taffon C., Foderaro S., Paccagnella E., Simonetti S. (2023). Network approach in liquidomics landscape. J. Exp. Clin. Cancer Res..

[170] Hashemi Gheinani A., Sack B.S., Bigger-Allen A., Thaker H., Atta H., Lambrinos G., Costa K., Doyle C., Gharaee-Kermani M., Patalano S. (2025). Multiomics analysis unveils an inosine-sensitive DNA damage response in neurogenic bladder after spinal cord injury. JCI Insight.

[171] Prabhu K.S., Kuttikrishnan S., Ahmad N., Habeeba U., Mariyam Z., Suleman M., Bhat A.A., Uddin S. (2024). H2AX: A key player in DNA damage response and a promising target for cancer therapy. Biomed. Pharmacother..

[172] Shang Y., Wang X., Su S., Ji F., Shao D., Duan C., Chen T., Liang C., Zhang D., Lu H. (2024). Identifying of immune-associated genes for assessing the obesity-associated risk to the offspring in maternal obesity: A bioinformatics and machine learning. CNS Neurosci. Ther..

[173] Zhang B., Liu H., Wu F., Ding Y., Wu J., Lu L., Bajpai A.K., Sang M., Wang X. (2024). Identification of hub genes and potential molecular mechanisms related to drug sensitivity in acute myeloid leukemia based on machine learning. Front. Pharmacol..

[174] Drew Y., Zenke F.T., Curtin N.J. (2025). DNA damage response inhibitors in cancer therapy: Lessons from the past, current status and future implications. Nat. Rev. Drug Discov..

[175] Atkinson J., Bezak E., Le H., Kempson I. (2024). DNA Double Strand Break and Response Fluorescent Assays: Choices and Interpretation. Int. J. Mol. Sci..

